# Identification of *Pseudocercospora mori* as the causal agent of grey leaf spot disease in mulberry (*Morus atropurpurea*) from various localities in Guangdong Province, China

**DOI:** 10.3389/fpls.2025.1648690

**Published:** 2025-09-04

**Authors:** Izhar Hyder Qazi, Ting Yuan, Xi Liu, Jiping Liu

**Affiliations:** Guangdong Provincial Key Lab of Agro-Animal Genomics and Molecular Breeding, College of Animal Science, South China Agricultural University, Guangzhou, Guangdong, China

**Keywords:** barcoding genes, high-throughput sequencing, leaf spot disease, mulberry, phylogenetic analysis, sericulture, sub-tropical

## Abstract

During periods of high temperature and humidity, mulberry trees become susceptible to fungal leaf spot disease, which can significantly reduce both the yield and quality of their leaves. In this study, we collected samples of mulberry leaf spot disease from six regions of Guangdong province of China. The disease samples were studied using traditional morphological methods, high-throughput sequencing technology, molecular phylogenetic analysis, and pathogenicity tests. The observed morphological features of the pathogen were consistent with those of *Pseudocercospora*. High-throughput sequencing results revealed the presence of multiple fungal species in the samples, with *Pseudocercospora* spp. comprising the highest proportion. The complete rDNA and mitochondrial genome sequences of *Pseudocercospora* spp. were assembled. Based on the sequencing data, primers were designed to amplify and sequence barcode gene regions, including *ITS*, *Cyt b*, and *COI*. Phylogenetic analyses consistently placed the pathogen within the family Mycosphaerellaceae. ITS-based identification confirmed the pathogen as a member of the genus *Pseudocercospora*, while the *Cyt b* and *COI* sequences indicated a relatively distant relationship with the closely related genus *Cercospora*, thereby supporting the morphological classification of the pathogen at the molecular level. In addition, pathogenicity validation identified *Pseudocercospora mori* as a primary causal pathogen of leaf spot disease in mulberry. PCR primers specifically designed based on the rDNA sequence of *Pseudocercospora mori* achieved a detection sensitivity as low as 3 × 10⁻² ng/μL. In conclusion, based on morphological and molecular phylogenetic evidence, we identified *Pseudocercospora mori* as the causal pathogen of mulberry leaf spot disease. This study provides useful data for practical management of mulberry leaf spot disease at the field level, aiding in the sustainable development of sericulture.

## Introduction

Mulberry is a perennial herbaceous plant native to China (Huang et al., 2025). It holds great economic importance in sericulture industry, as mulberry leaves are used as the only feed resource of the domesticated silkworms (Ji et al., 2008; Chan et al., 2016; Nguyen et al., 2025). Due to the presence of essential flavonoids, oligosaccharides, proteins, and volatile compounds, mulberry and its byproducts are commonly used in human and animal medicine and food/feed industries (Bai et al., 2024; Deng et al., 2024; Lin et al., 2025; Hu et al., 2024; Mao et al., 2025; Zhang et al., 2024; Guo et al., 2025; Hou et al., 2024; Xie et al., 2025). Due to their above mentioned versatile medicinal and nutritional utilities, mulberry and its byproducts generate economy worth billions of yuan (CNY) annually, supporting China’s silk industry, allied businesses, and supply chains (Liao and Xiao, 2013). However, the stable development and growth of sericulture and mulberry production systems continue to be affected due to several devastating diseases. For instance, during hot and humid seasons, mulberry trees are susceptible to fungal diseases (Luo et al., 2024). From these, the fungal leaf spot disease (also known as leaf stain or blight) is one of the important diseases affecting mulberry production in China and other sericulture-intensive countries. The causal pathogens of fungal leaf spot disease of mulberry are complex and diverse, with varying occurrence and severity of the disease in different parts of the world (Arunakumar et al., 2023; Saif et al., 2023; da Costa et al., 2021; Soylu et al., 2003). Although, the disease is caused by many different fungi, the fungal leaf spot disease often exhibits similar pattern and cycles. Importantly, the disease significantly affects the mulberry leaf quality and nutritional value (Arunakumar et al., 2023).

The grey leaf spot, caused by *Pseudocercospora mori*, is recognized as one of the most widespread mulberry diseases in China, severely impacting the leaf quality and yield of mulberry (Zhang, 1975; Mekala et al., 2025). Although the disease causes significant economic losses, little information is available about its distribution and occurrence in China. In addition, the exact estimation of its economic impact is difficult due to the complexities of farming practices and sericulture industry (Liu Jiping, personal communication).

The grey leaf spot disease is typically characterized by dark, mold-like spots and necrotic lesions on the undersides of leaves, leading to reduced leaf quality and premature leaf drop, which significantly affects sericulture and the medicinal and nutritional value of mulberry leaves (Teotia et al., 1997; Shree et al., 1997). The disease reduces chlorophyll, sugars, and proteins in leaves while increasing phenolic compounds, impairing photosynthesis and water use efficiency (Kumar et al., 2011a; Shree et al., 1997). The disease has been reported in Australia (Grice et al., 2006), Iran (Hesami et al., 2012), India (Govindaiah et al., 2002), Pakistan (Niaz et al., 2010), Thailand (Phengsintham et al., 2013), and Kenya (Peris et al., 2012).

*Pseudocercospora mori* primarily overwinters as mycelium in infected leaf tissues, with overwintering conidia having low viability in natural conditions are insufficient to cause an infection. The following year, overwintering mycelium produces conidia that spread and initiate the primary infection, followed by secondary conidia from new lesions, leading to widespread disease (Zhang, 1975; Wang et al., 1994; Babu et al., 2002). It is believed that both pathogen accumulation and environmental influences contribute to the intensification of the disease (Huang and Zhu, 2013). Located in the subtropical climate zone with ample sunlight and rainfall, Guangdong province is one of the important sericulture and mulberry production areas in China. Mulberry trees are typically densely planted as shrubs, creating favorable conditions for grey leaf spot outbreaks, resulting in substantial losses to the farmers and industry (Govindaiah et al., 2002; Maji et al., 2008). Based on the traditional morphological analysis methods, *Pseudocercospora mori* is classified in the Deuteromycotina subphylum, with well-developed mycelium producing scattered conidia on mycelium or conidiophores, lacking sexual reproductive characteristics, and belonging to the Dematiaceae family (Qiu, 1998; Ávila et al., 2005). However, influenced by the notion that “sericulture outweighs mulberry cultivation,” the knowledge of this pathogen remains limited locally and globally, hindering effective identification and control of grey leaf spot disease (Zhang, 1975).

Recent technological advancement has helped in molecular identification of novel plant pathogens based on their DNA sequence analysis. The ITS sequences, rRNA sequences, and mitochondrial genes (e.g., *COI*, *Cyt b*) are targeted for rapid fungal identification (Crous et al., 2013a, Crous et al., 2013b; Schoch et al., 2012; White et al., 1990; Hebert et al., 2003; Stielow et al., 2015; Gao and Zhang, 2013). ITS sequences, with conserved flanking regions and moderate variability, are the primary barcode for fungal identification but they require integration with the host and morphological data due to high interspecies similarity or plant DNA interference (Schoch et al., 2012; White et al., 1990). High-throughput sequencing enhances identification accuracy, supporting population dynamics and gene function studies (Walkowiak et al., 2016). Disease epidemiology is influenced by pruning timing, shoot age, and weather conditions, yet these relationships remain underexplored (Kumar et al., 2011a; Maji et al., 2008; Hawksworth, 2003).

Given the economic value of mulberry leaves and the significant threat of the grey leaf spot disease in Guangdong province of China, this study integrated traditional morphological, high-throughput sequencing, and molecular analyses to systematically investigate the symptoms, describe pathogen morphology, and identify the locally prevalent causal agent of grey leaf spot disease of mulberry (*Morus atropurpurea*).

## Materials and methods

### Material

**Figure 1:**
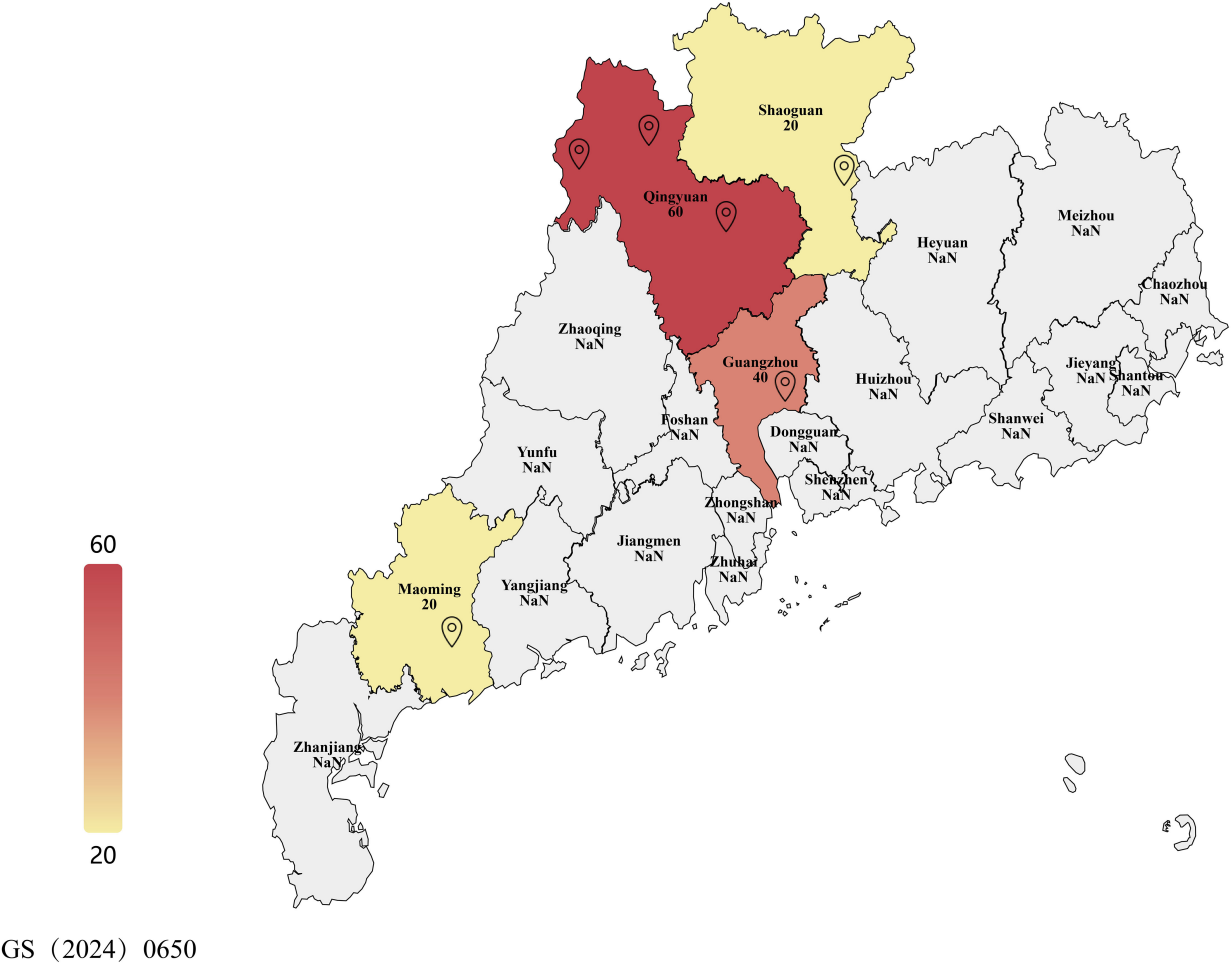
Map showing sampling locations across Guangdong province and number of samples of leaf spot-infected mulberry leaves. This map shows the administrative divisions of Guangdong Province, China. Landmarks represent sampling locations in this study. Samples were collected in six locations across three cities: Guangzhou City, Shaoguan City, Qingyuan City, and Maoming City. Color intensity represents the number of samples collected. For further details, readers can refer to **Table 1**.

The main biological materials in this study were mulberry (*Morus atropurpurea*; Guisangyou 12 variety) leaves affected with leaf spot disease (see **Figure 1** and **Table 1**). Mulberry leaves showing typical symptoms of leaf spot disease were collected from six mulberry orchards in Guangdong Province of China. Infected leaves had jet-black to dark gray lesions that were round, irregular, or oval in shape, measuring 2–10 mm in diameter. Initially, they appeared as small, dark spots that gradually expanded and merged into larger patches. These lesions were primarily located in the lower middle side or margins of leaves. The detailed description of collected material is given in **Table 1**. Briefly, the material was collected from different localities of Guangdong province including Guangzhou City, Wengyuan county, Shaoguan city, Yangshan county, Qingyuan city, Liannan Yao Autonomous county, Qingyuan city, Yingde city, Qingyuan city, Huazhou city, and Maoming city. In addition, winter soil and branch materials were collected from Yingde city, Qingyuan city, Huazhou and Mulberry demonstration base of the South China Agricultural University, Guangzhou, China.

**Table 1:** Number and collection location of mulberry leaf spot disease leaves.

S. no.	Collection area	Geographic location (east longitude, north latitude)	Number of samples
1	Guangzhou City, Guangdong Province (GZ)	113.359528, 23.170115	40
2	Wengyuan County, Shaoguan City, Guangdong Province (WY)	114.143277, 24.369938	20
3	Yangshan County, Qingyuan City, Guangdong Province (YS)	112.639911, 24.471893	20
4	Liannan Yao Autonomous County, Qingyuan City, Guangdong Province (LN)	112.039816, 24.548602	20
5	Yingde City, Qingyuan City, Guangdong Province (YD)	113.457986, 24.184965	20
6	Huazhou City, Maoming City, Guangdong Province, China (HZ)	110.654611, 21.664831	20

Cladosporium sp., Penicillium verruculosum, Aspergillus sp., Phanerina mellea, Schizophyllum commune, Lasiodiplodia theobromae, Candida mucifera, Phyllactinia moricola, Ciboria canrunculoides, Lecanicillium psalliotae and Pseudomonas aeruginosa were obtained from the Regional Sericulture Training Center for the Asia-Pacific, College of Animal Science, South China Agriculture University, Guangzhou, China.

#### Identification of causal fungal pathogen of leaf spot disease based on traditional morphological analysis

Mycelium, conidial peduncle, conidia, ascospores, spore producing cells, etc. were photographed and recorded, and microscopic orders of magnitude such as mycelium width, conidium size, conidial peduncle length and width, and the number of conidial septa were described and recorded in detail (Woudenberg et al., 2013). A fluorescence microscope MF30 (Guangzhou Mingmei Optoelectronics Technology Co., Ltd.) was used to obtain photographs. A ruler was added using the Mshot Image Analysis System of Mingmei Microscopic Digital Measurement and Analysis System. The length and width of conidia, width of hyphae, length and width of hyphal peduncle, and size of the septa were measured and the relevant data were counted. The mycelium and conidia of the fungi were stained and observed using dyes such as Calcofluor White Stain, Fluorescent Brightener 28 Stain, and AOPI Stain. Morphological descriptions of several possible genera of mulberry leaf spot pathogens belonging to the family Dematiaceae (dark spore family) were compared in an attempt to identify the genera of the pathogens.

#### Molecular identification of causal fungal pathogen of leaf spot disease

In this study, typical samples of grey leaf spot disease of mulberry (*Morus atropurpurea*; Guisangyou 12 variety) collected from Guangzhou, Guangdong Province were observed under a microscope to ensure that the diseased samples contained abundant target pathogen spores and very few other pathogens/microorganisms.

### DNA extraction and PCR amplification

The extraction of fungal genomic DNA was based on the CTAB method and/or as per the instructions of Genview GV-Filamentous Fungi Genomic DNA Extraction Kit (LOT: 61014010105). The extraction of DNA of mulberry leaves was performed using Plant Genomic DNA Extraction Kit (LOT: 69700110). The bacterial DNA (where applicable) was extracted using Shanghai Bio-Tech SanPrep Column Plasmid DNA Mini-Extraction Kit. The DNA from soil collected from around the morbid mulberry trees was extracted using the Genview kit with some modifications.

PCR was performed using a 2×Taq Master Mix from Microanalysis Co., Ltd., containing Taq DNA polymerase, Tris-HCl, (NH_4_)_2_SO_4_, MgCl_2_, and dNTPs. Primers at 0.4 μmol/L were provided by Sangon Biotech (Shanghai) Co., Ltd., Guangzhou Branch. Annealing temperatures were adjusted based on melting temperatures of primers. The PCR protocol included an initial denaturation at 94°C for 5 minutes, 30 cycles of 94°C for 30 seconds, annealing at 55-58°C for 30 seconds, and 72°C for 1 minute, ending with a final extension at 72°C for 10 minutes. PCR products (3–5 μL) were analyzed on 1.4% agarose gels stained with ethidium bromide and visualized with the Tanon-1200 system. Target fragments were purified using a DNA kit, involving gel dissolution, spin column processing, and ethanol washes, then eluted and stored at -20°C. Sequencing was done by Sangon Biotech, assembled with Lasergene Seqman, and compared via NCBI BLAST for species identification.

Phylogenetic analyses (where applicable) were performed using the Maximum Likelihood (ML) method in MEGA12 software (Kumar et al., 2024).

### High-throughput sequencing of mulberry leaf spot disease pathogens

The diseased leaves with relatively single colonies were selected and sent to Guangzhou Ruike Gene Technology Co. Ltd. for high-throughput sequencing of the whole genome of ribosomal DNA (rDNA) and mitochondrial DNA (mtDNA). The presence of fungi in the colonies was analyzed through the assembly and annotation of the sequencing results. The full length of the sequences of rDNA and mDNA of the major pathogenic fungi were also obtained.

### DNA extraction, library construction and sequencing

The total DNA was extracted using the Fungal DNA Extraction Kit following the manufacturer’s instructions, and the extracted DNA was stored at -20°C. The total DNA was constructed into a double-ended, high-throughput sequencing library with a 500-bp insertion using the Illumina Hiseq2500/4000, and a total of 4.38 Gb of high-throughput sequencing was generated.

### Gene sequence assembly

As the DNA extraction process included leaf material, therefore, in order to reduce the influence of the mulberry genomic data on the microbial sequence assembly, the genomic DNA sequences of the mulberry leaves were firstly removed before performing the microbial sequence assembly. The whole genome sequence of *Morus notabilis* (GCA_000414095.2) and the chloroplast genome sequence (NC_027110.1) were selected as the reference genome sequences. The data were compared and analyzed using the BWA (0.7.12-r1039) mapping software. To map the sequencing data with the reference genome of mulberry tree and to determine the sequenced fragments of the reference genome in comparison with genome sequence of mulberry tree, MEM comparison algorithm was selected using the double-end comparison method, with default parameters of the software. A computer program written in python was used to remove the mulberry sequencing data from the fastq sequencing data before proceeding to microbial sequence assembly. Microbial sequence assembly was performed using MetaVelvet (v1.2.01) assembly software.

### Sequence annotation, species identification and species abundance analysis

The sequence tag annotation was performed using blastn (2.2.31+) sequence comparison analysis software. The assembled sequence tag sequences were compared with the nt database of the NCBI. The blastn comparison was set to an expectation value of <1e-20, and the sequence tags were annotated based on the comparison results. The rDNA sequences are important and most commonly used molecular markers for bacterial and fungal identification, so species taxonomic identification and quantification use rDNA as the main molecular marker. Therefore, based on the results of sequence tag annotation, rDNA sequences were selected as the basis for microbial identification and quantitative analysis. Using BWA (0.7.12-r1039) + samtools (v1.2) analysis software, the average sequencing depth of rDNA fragments in the sequencing data was calculated and used as the abundance value of the species.

### Complete ribosomal DNA assembly and experimental validation

The rDNA of fungi consists of 18S segment, ITS1 segment, 5.8S segment, ITS2 segment and 28S segment, with a total length of the sequence about 6 Kb. The initial sequence tag assembled by MetaVelvet (v1.2.01) was a broken ribosomal tag. Therefore, in order to obtain complete rDNA sequences, the analysis was performed using the sequence capture and *de novo* assembly strategy. The rDNA sequence containing the ITS sequence of the target pathogen was selected as the reference sequence, and BWA (0.7.12-r1039) software was used to perform 0 mismatch and 0 gap comparison. Based on the comparison results, the double-ended sequenced fragments were obtained from the sequencing data. The sequence was further assembled and extended using MetaVelvet (v1.2.01) assembly software. The complete rDNA sequence was obtained after multiple cycles.

### Annotation and GC ratio analysis of ribosomal DNA sequences

The rDNA sequences were annotated using blastn (https://blast.ncbi.nlm.nih.gov/) software of the NCBI to compare the rDNA sequences with the nt database, and 28S region, ITS1 region, 5.8S region, ITS2 region and 28S region were annotated. The GC ratios of the sequences were analyzed using a computer program written in python language to calculate the average GC ratio of each region. The program calculates the GC ratio characteristics of rDNA with a window of 100bp and step size of 10bp.

### Calculation of genetic distances and systematic classification

The 18S sequences, ITS1 + 5.8S+ITS2 sequences, 28S sequences, and complete rDNA sequences were compared with closely related species using MUSCLE (v3.8.31) package. Genetic distances between each segment of the rDNA sequence and the relatives were calculated separately using MEGA 12 software. The optimal alternative model for the phylogenetic tree was selected using jModelTest2 (https://github.com/ddarriba/jmodeltest2) and this was substituted into the phylogenetic tree constructed using the maximum likelihood (ML) method, with 1,000 iterations of the evolutionary tree.

### Molecular identification of pathogenic fungi based on DNA barcoding

The validation and specific detection primers for the ITS region were designed based on the complete rDNA sequence results. Based on the whole genome sequence annotation results of mtDNA, the validation and specific detection primers were designed for the amplification of partial regions of *Cyt b* and *CO I* genes. These primers were validated and used for pathogen-related gene sequence differences in different regions. Primer Premier 5.0 and NCBI Primer BLAST software were used to design primers. For PCR amplification conditions and procedures, see sub-section “*DNA extraction and PCR amplification*”.

The amplified sequences were compared using the BLAST function of the NCBI, taking into account Identities and Query cover, and judged according to the score in ascending (high to low) order to determine the closest species/strains. The phylogenetic analysis was performed and trees were constructed using MEGA12 software.

### Isolation and purification of fungal spores from mulberry leaves

Initial attempts to isolate the fungal pathogen on Potato Dextrose Agar (PDA) were unsuccessful. As a result, we employed a serial inoculation technique using fresh mulberry (*Morus atropurpurea*) leaves, and the isolate’s purity was confirmed through microscopic examination. Symptomatic leaves were rinsed, surface-sterilized with 1% sodium hypochlorite for 60 s, rinsed thrice in sterile water, and blotted dried. Infected tissue was excised, suspended in 300μL sterile water, vortexed for 1 min, and filtered through cheesecloth to yield a spore suspension (10^4–^10^5^ spores/mL). Healthy mulberry leaves were sterilized and placed in a double-layer petri dish system, with petioles accessing water through 5-mm holes. The suspension was diluted (1:100) and inoculated (10 μL, 4–6 sites/leaf) onto leaves, incubated at 22–25°C with a 12-h photoperiod for 7–14 days. Spores from resulting lesions were harvested with 300 μL water containing 0.01% Tween 80, filtered (40-μm mesh), and re-inoculated onto new leaves for three cycles. Lesions were examined (400× magnification) to confirm spore uniformity and purity. Final spores were harvested, centrifuged at 3,000 × g for 5 min, and resuspended to 10^5–^10^6^ spores/mL. Spores were stored at 4°C (short-term) or −80°C in 20% glycerol (long-term). Pathogenicity was confirmed by inoculating spores onto healthy leaves to fulfill Koch’s postulates, and DNA extraction for molecular identification (Choi et al., 1999; Agrios, 2005).

### Re-inoculation experiment for verification of pathogens based on Koch’s postulates

In this experiment, the conidia of the mulberry leaf spot disease pathogen were collected and used as a test material. The inoculum was prepared by eluting the conidia in sterile water and shacked evenly. The density of conidia suspension was adjusted to a concentration of 1.0 x10^8^ spores/mL (Babu et al., 2002). Detached healthy and mature mulberry leaves were used for inoculation. The conidia suspension was applied to the back of the mulberry leaves with sterile absorbent cotton dipped in the conidia suspension. The process was repeated several times, and then the material was sealed in sterile bags, and a certain amount of spore suspension was sprayed with a spray bottle before final sealing. At an interval of 10 days, the inoculated leaves were randomly picked and observed to check pathogen infection (to check whether the pathogen infection has entered the latent stage). If conidia hyphae were observed on the leaves, they were deemed suitable for downstream analysis. The lesion part of the grafted sample was observed microscopically, and the morphological characteristics of the lesion were recorded and compared with the lesion under natural conditions to determine whether the disease state of the grafted sample was consistent with the natural disease state.

### Detection of pathogenic fungi

The mycelia on diseased mulberry leaves were eluted and pure DNA of the pathogen was extracted. Mulberry leaves were collected from plants at different stages of the disease, including the early, peak and late stages. The same weight of leaves was weighed for all disease stages and used for the DNA extraction. The leaves collected at the onset (early) of the disease were treated differently to simulate the degradation process of plant leaves in the outdoor environment, including aging, air-drying and rotting treatments. In addition, the DNA was extracted from the soil around the morbid mulberry leaves in the mulberry orchard and the epidermis of the morbid mulberry branches. The specific primers were designed and used to verify the presence of the leaf spot disease pathogen in the samples.

In the re-inoculation experiment for the early stage of infection, the pathogenic fungus invaded into the mesophyll cells of mulberry leaves but no large number of mycelia, i.e., visible spot and lesions were formed on the surface of the mulberry leaves. The situation was similar to that of the leaves before the outbreak of the disease. The DNA was extracted from the leaves at the early stage of infection, and then subjected to standard PCR amplification and electrophoresis to observe the presence of the target bands and to confirm the infection.

## Results

### Observation and comparison of symptoms of mulberry leaf spot disease in different regions of Guangdong Province of China

**Figure 2:**
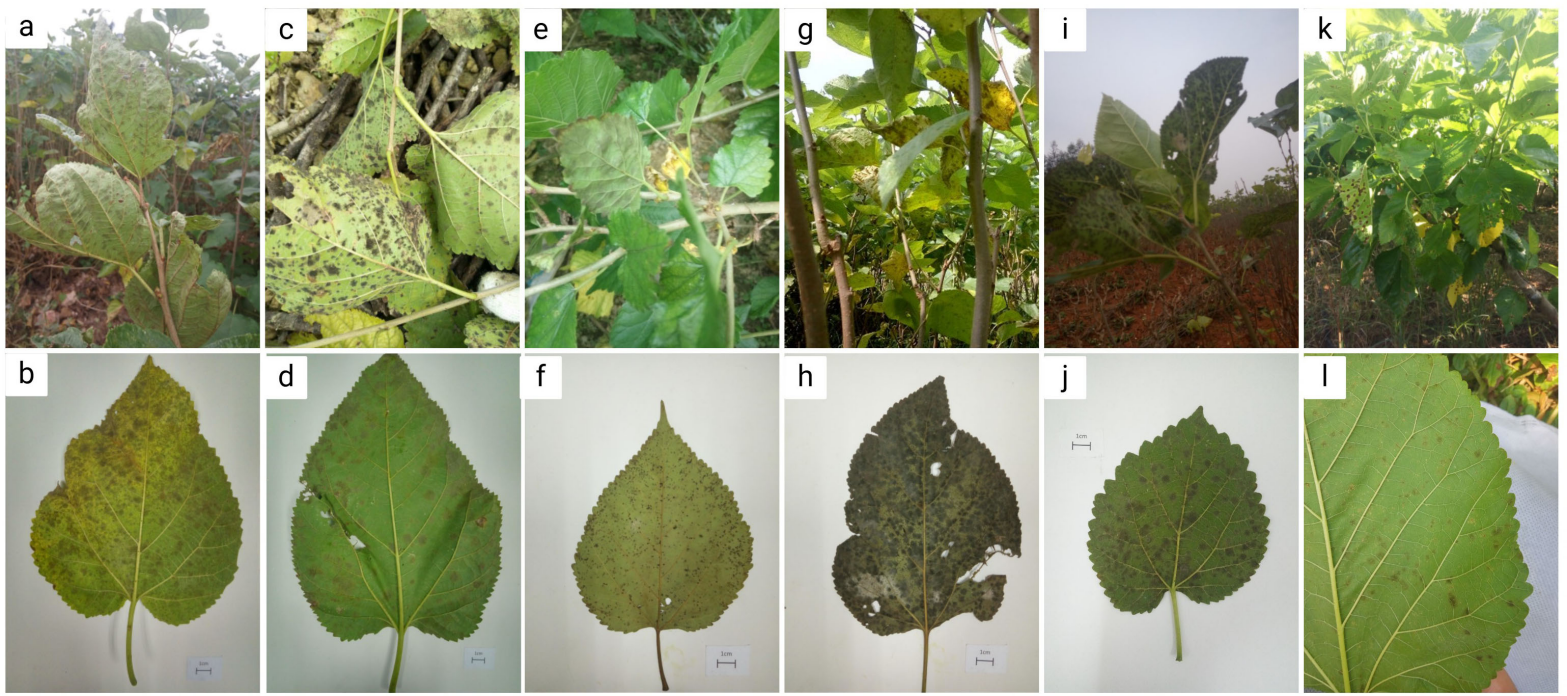
Symptoms of mulberry leaf spot disease in samples collected from different areas of Guangdong Province. **(a, b)** are diseased leaves collected from mulberry demonstration base of South China Agricultural University, Guangzhou city. **(c, d)** are diseased leaves collected from mulberry orchard of silkworm farmers in Wengyuan county, Shaoguan city. **(e, f)** are diseased leaves collected from mulberry orchard at the silkworm extension center of Yangshan county, Qingyuan city. **(g, h)** are diseased leaves collected from mulberry orchard at the silkworm extension center of Liannan Yao Autonomous county, Qingyuan city. **(i, j)** are diseased leaves collected from mulberry orchard at Yingde silkworm seed farm, Yingde city, Qingyuan city. **(k, l)** are diseased leaves collected from mulberry orchard at the silkworm extension center of Huazhou city, Maoming city.

As shown in **Figure 2**, the manifestation of mulberry leaf spot disease in the samples collected from six cities or counties in Guangdong Province were different in different regions and collection periods. In Guangzhou, the samples were collected during the transition from the summer and autumn to early winter, over a time period of three months. During this period, the mulberry leaf disease gradually spread from the middle and lower parts of the branches to the whole plant, and even to the top of the branches. The number of spots on each leaf was higher, and the spots were in the form of grey mold, indicating abundant mycelia. In the late stage plantation, the land was relatively dry, and the aging degree of the leaves was higher, and the diseased leaves could be seen at the top of the branches (**Figures 2a, b**). In the samples collected from Wengyuan county, the leaf spots were black, with fewer mycelia. The spots appeared like dye, and fewer discolored spots were observed on the leaf surface (**Figures 2c, d**). The samples from Yangshan county were collected in the early autumn. The management of the mulberry plantation was better, with minimal incidence of the mulberry leaf spot disease. The samples were collected from mulberry trees maintained in a low shrub form, with leaf-bearing branches located close to the ground, and the back of the leaves showing a lighter black powdery appearance (**Figures 2e, f**). In Liannan County, the samples were collected from middle of the branches mulberry trees planted in the form of shrubs. The appearance of leaves was poor with grey spots, and no discoloration spots on the leaf surfaces. At the time of sampling, there were milder episodes of mulberry leaf spot disease (**Figures 2g, h**). The samples were collected from Yingde City in the winter, and the episodes of mulberry leaf spot disease were serious. The lesions of mulberry leaf spot disease were black and almost covered the back of the leaves, but there were not many discolored spots on the leaf surface (**Figures 2i, j**). The samples from Huazhou city were collected in the summer, when the attack of mulberry leaf spot disease was mild. The samples were collected from the lower part of the branches, with lighter characteristics of the disease, a lighter degree of grey coloration, and a lesser number of leaves affected with lower number of spots on the leaves (**Figures 2k, l**).

### Pathogen identification based on morphological observation after host-based purification of mulberry leaf spot pathogen spores

**Figure 3:**
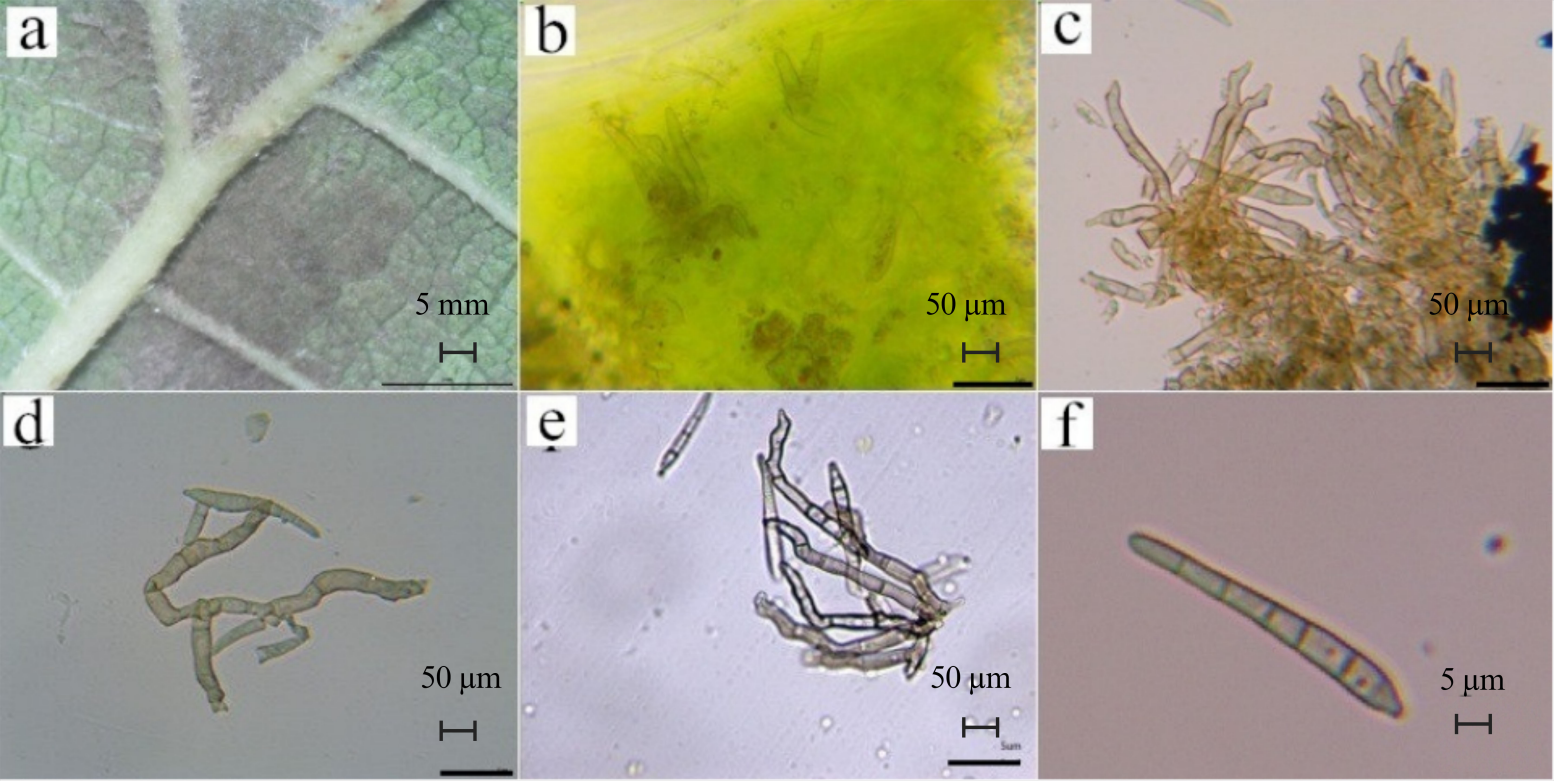
Morphological observations of pathogenic fungi of leaf spot disease. **(a)**. epiphytic mycelium (on the back of the leaf; scale bar is 5 mm); **(b)**. endophytic mycelium (inside the leaf), **(c)**. developed ascospores, **(d)**. mycelium, **(e)**. conidiophores on the undeveloped ascospores, (scale bar is 50 μm); **(f)**. Mature septate conidia (scale bar is 5 μm). These images are obtained after host-based purification of mulberry leaf spot pathogen spores.

**Figure 4:**
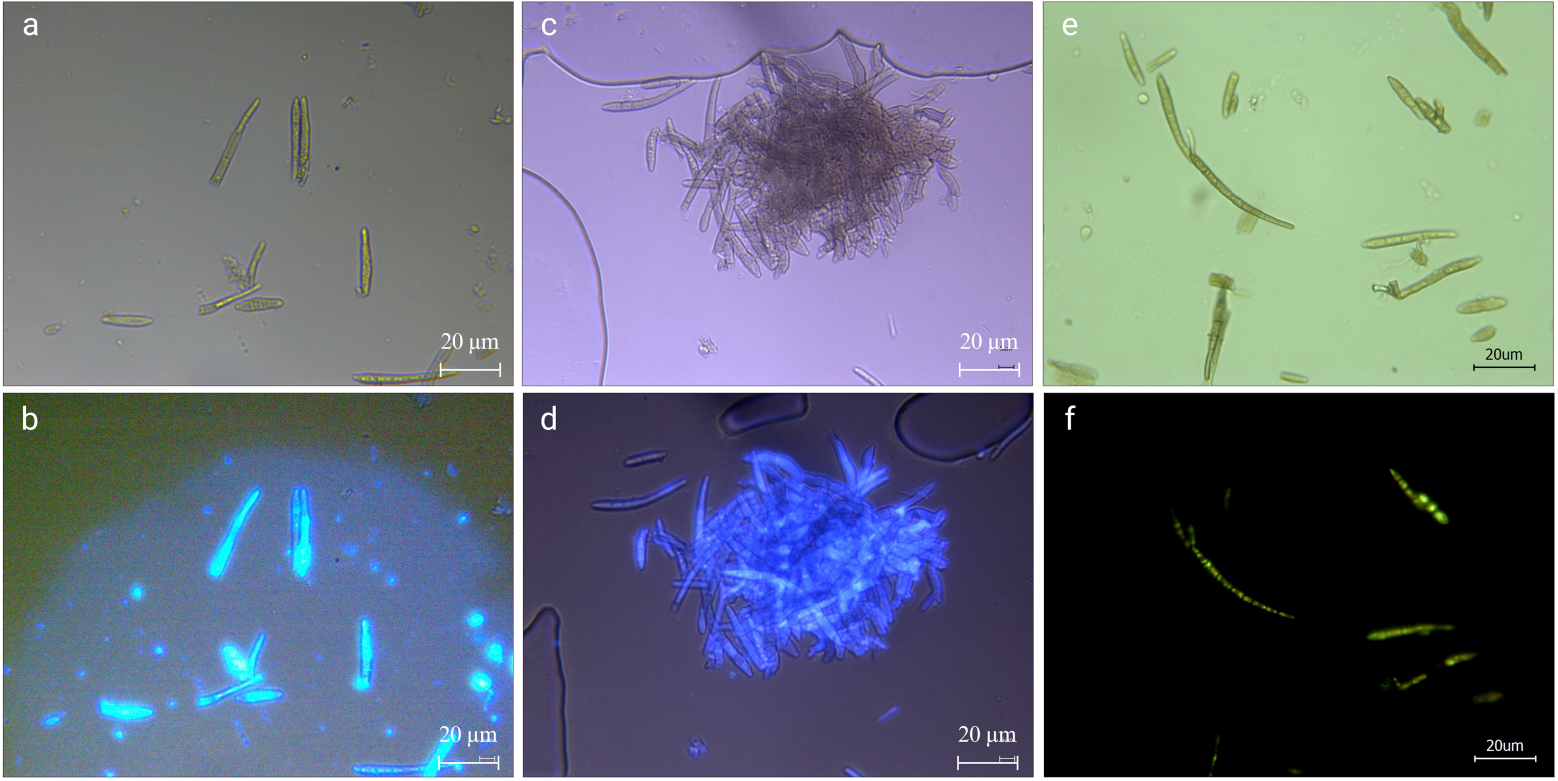
Calcofluor white stain **(a, b)**, fluorescent brightener 28 **(c, d)**, and AOPI **(e, f)** staining of pathogenic fungi. **(a, c)** are conidia under the normal light field after staining; **(b)** is the conidia in the UV light field after Calcofluor White Stain staining; **(d)** is the conidia in the UV light field after Fluorescent Brightener 28 staining. **(e)** is hyphae and conidia under normal light after staining; **(f)** is hyphae and conidia under blue light after staining. The scale bar is 20 μm.

The samples of mulberry leaf spot disease collected from six areas in Guangdong Province were scraped and diluted for observation of morphology of the pathogen under the microscope. Host-based purification via three serial leaf inoculations ensured pathogen purity. The representative images are shown in shown in **Figures 3**, **4**, and **Supplementary Figure 1**. The microscopic observation revealed that the longer conidia had six septa crumpling inwards. The widest part of the conidia was 5.233 μm and the length reached 53.410 μm. Based on length and number of septa, it was determined that these were the conidia produced on the hyphae. The shorter conidia had three septa crumpling inwards. The widest part of the conidia was 5.436 μm, and the length reached 26.220 μm. Based on length and number of septa, it was determined that the conidia were produced on the conidiophore.

As shown in **Figure 3**, the mycelia were found to be epiphytic (found on the external surface of leaves; **Figure 3a**) and endophytic (found inside the leaves; **Figure 3b**), with well-developed (**Figure 3c**) or smaller ascospores (**Figure 3d**). According to the morphological identification methods of related fungi, there were conidiophores on the ascospores, which were clustered (**Figure 3e**), zigzag, irregular in shape, and partially branched, so *Clasterosporium* was ruled out. When the conidia are produced, they are produced in the form of buds, and after falling, the spore-producing cells can continue to grow and swell, which is a full-wall budding growth. Our morphological observation revealed that the conidia only had septa (**Figure 3f**), which were relatively straight stick-shaped, and the morphology didn’t conform to the description of *Sirosporium*. The conidia were produced on hyphae, and the hyphae were endophytic or epiphytic, so *Cercospora* was ruled out. Based on foregoing typical morphological characteristics, the pathogen of mulberry leaf disease was presumed to be *Pseudocercospora*.

As shown in **Figures 4a, b**, the pathogens produced a higher brightness when stained with the Calcofluor White, indicating that their cell wall contained cellulose or chitin, which combined well with the stain. Using fluorescent colorimetry, it was found that, in addition to mycelium and conidia, more colorable impurities can be seen (**Figure 4b**), most of those were the impurities that cannot be seen in the ordinary light field (**Figure 4a**). The Fluorescent Brightener 28 stained the pathogen uniformly. Under the UV light, the fluorescence brightness was weaker than that of Calcofluor staining, but the staining was more uniform (**Figures 4c, d**). The staining effect of impurities was also weaker, indicating that the stain didn’t affect the observation of fungi and that the fungal conidia contained chitin.

### AOPI staining to determine the activity of pathogens

The conidia stored at 4°C for 60 days to simulate overwintering were stained with AOPI to test the viability of conidia when stored at low temperature for a longer period of time. As shown in **Figures 4e, f**, except for a few, most of the conidia were stained in each field of view, (**Figure 4e**). The hyphae were weakly stained and some sections of conidia were orange-red, but there were complete green conidia (**Figure 4f**), indicating that there were still many viable conidia. However, the base number of overwintering conidia was large, and even if the survival rate was very low, the possible infection cannot be underestimated.

#### Analysis and application of high-throughput sequencing results

### Sequencing data analysis and sequence assembly

Sequencing was performed using the Illumina Hiseq2500/4000 high-throughput sequencing platform, and a total of 18.56 M pairs of sequenced fragments were sequenced, with a double-ended 125 bp read length and a total sequencing data of 4.64 Gb. After sequence alignment with *Morus notabilis* whole genome sequence (GCA_000414095.2) and chloroplast genome sequence (NC_027110.1), a total of 17.11 M (4.27 Gb) sequenced fragments were aligned with the reference genome, which accounted for 92.14% of the total sequence data. The size of the remaining sequence data was 364.95 Mb, accounting for 7.86% of the total sequence data. The high-throughput sequencing data were assembled using MetaVelvet (v1.2.01), and a total of 304946 sequence tags were assembled.

#### Sequence annotation, species identification and species abundance analysis

**Figure 5:**
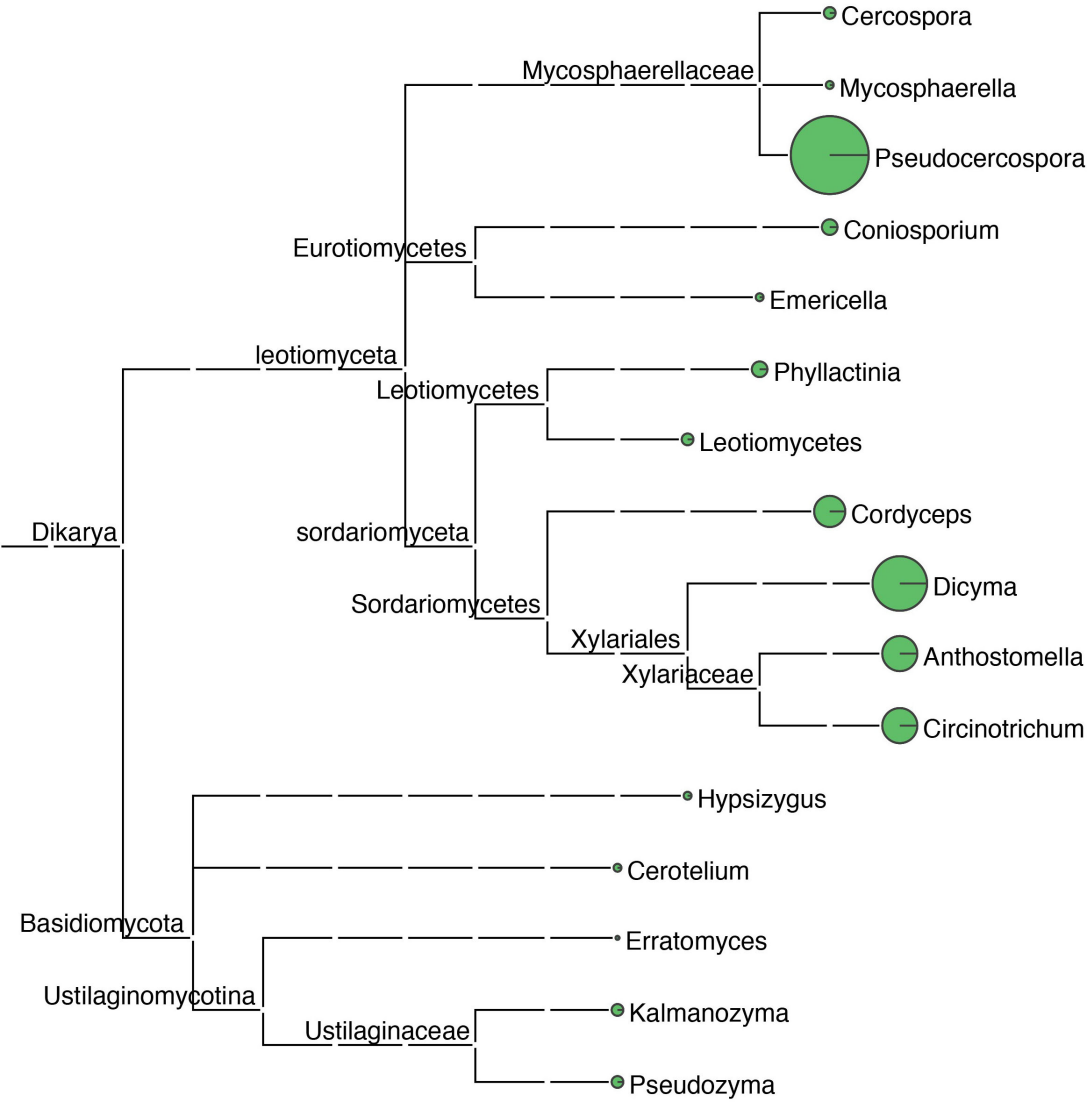
Taxonomic tree of fungal microorganisms identified in samples of leaf spot disease of mulberry. Using blastn (v2.5.0+) alignment analysis, sequenced ribosomal DNA sequence tags were aligned with the NCBI nt database, the Environmental Microbiology Database, and the Ricogene Pathogen Database. Based on multiple metrics, including sequence similarity, alignment length, and sequence integrity, a similarity assessment algorithm was used to select the optimal alignment to annotate the tag sequences and analyze the microbial diversity in the tissues. Sequence tag statistics were used to calculate the average sequencing depth of the sequence tags, which was used for quantitative microbial analysis. A plot of the microbial species and numbers was generated using MEGAN (MetaGenome Analyzer; Version 11). In this phylogenetic tree, the size of the circles at each taxonomic level reflects the abundance of the corresponding taxa, with larger circles denoting greater abundance and smaller circles indicating lower abundance.

Sequence tags were annotated using blastn (2.2.31+) and the nt database. A total of 261 rRNA sequence tags were annotated. By querying the sequence tag annotation results, a total of 16 genera of fungal microorganisms were found to exist on the leaf surface. The highest relative abundance was found in the genus *Pseudocercospora*, whose molecular tags were sequenced to a depth of 124X, with a relative abundance of 25.10%, accounting for the highest percentage and was identified as the main fungal pathogen in samples of mulberry leaf spot disease (**Figure 5**; **Table 2**).

**Table 2:** Fungi identified by annotation of high-throughput sequencing results.

Genus	Quantities	Percentage (%)
*Cercospora*	9	1.84
*Mycosphaerella*	4	0.82
***Pseudocercospora***	**123**	**25.10**
*Coniosporium*	18	3.67
*Emericella*	4	0.82
*Phyllactinia*	7	1.43
*Leotiomycetes*	6	1.22
*Cordyceps*	55	11.22
*Dicyma*	86	17.55
*Anthostomella*	69	14.08
*Circinotrichum*	76	15.51
*Hypsizygus*	6	1.22
*Cerotelium*	5	1.02
*Erratomyces*	1	0.20
*Kalmanozyma*	10	2.04
*Pseudozyma*	11	2.24
Total	490	100

The bold font is used to highlight the abundance of Pseudocercospora in the tested samples.

### Annotation and GC ratio analysis of rDNA sequence

Based on the results of high-throughput sequencing data assembly and experimental validation, the complete rDNA sequence of *Pseudocercospora* was assembled, with a length of 5469 bp (GC ratio of 50.67%) (**Supplementary Figure 2**). The sequence contained the 18S, ITS1, 5.8S, ITS2, and 28S regions. The 18S region was 1726 bp long (GC ratio 48.73%), ITS1 region was 150 bp long (GC ratio 58.00%), 5.8S region was 158 bp long (GC ratio 44.30%), ITS2 region was 149bp long (GC ratio 57.05%), and 28S region was 3286 bp long (GC ratio 51.37%) (**Table 3**; **Supplementary Figure 3**). The 18S and 28S regions were longer, accounting for 91.64% of the total length of the sequence. While in terms of GC proportion, the average GC proportion of ITS1 (58.00%) and ITS2 (57.05%) region was significantly higher than that of other regions (**Table 3**; **Supplementary Figure 3**).

**Table 3:** Full sequence annotation of rDNA of *Pseudocercospora*.

Region	Start (bp)	End (bp)	Length (bp)
18S rRNA	333	2059	1726
ITS1	2060	2225	165
5.8S rRNA	2226	2366	140
ITS2	2367	2516	149
28S rRNA	2517	5815	3298

#### Calculation of genetic distances and phylogenetic classification

**Figure 6:**
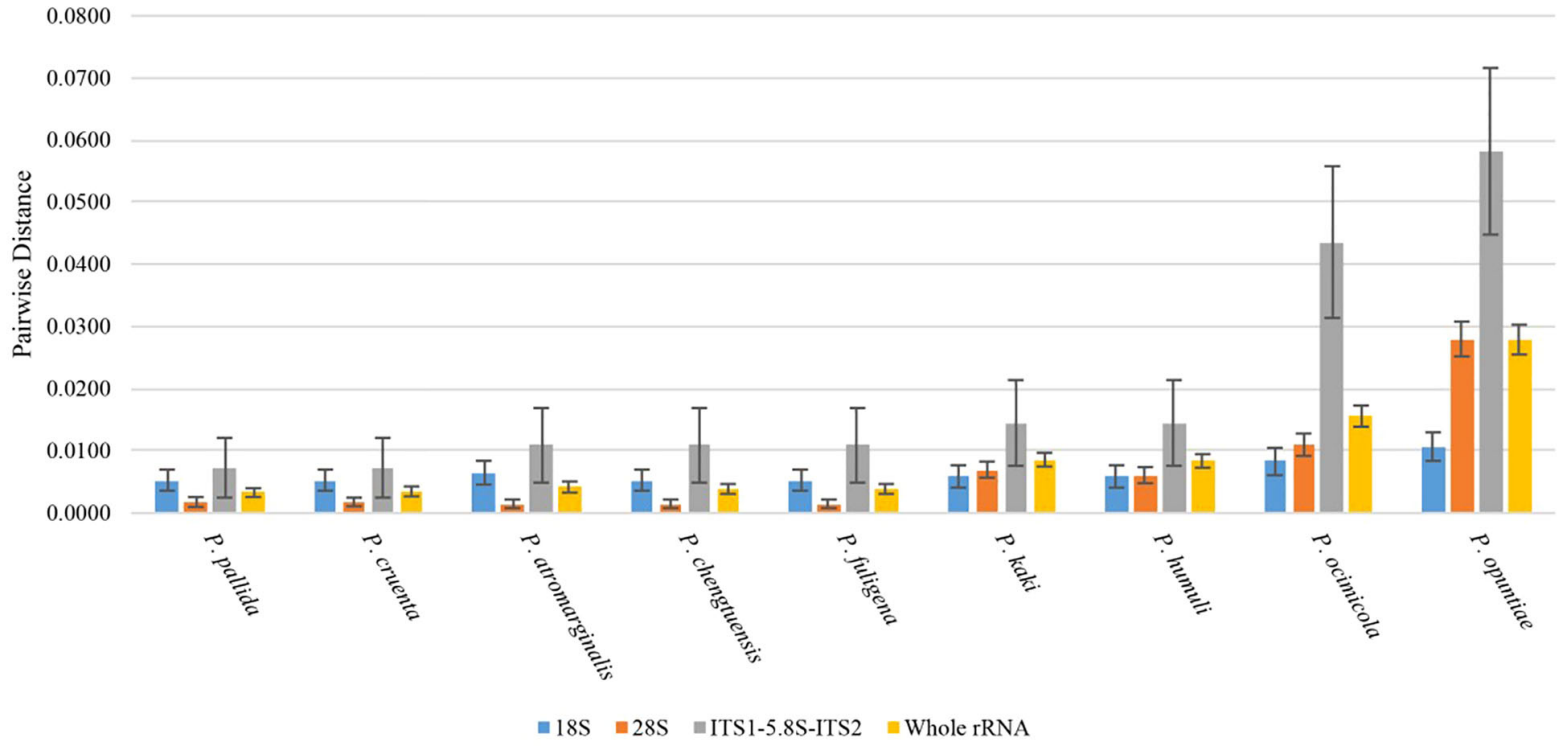
Plot of genetic distance analysis (height of bar graph is p-distance, whisker line is standard deviation).

The p-distance between species of the genus *Pseudocercospora* and the mulberry leaf pathogen *Pseudocercospora mori* GD in the 18S, ITS1 + 5.8S+ITS2, and 28S regions and the intact rDNA was calculated using MEGA12 (**Figure 6**). The 18S sequences were highly conserved between species with little variation between species. The ITS sequences, although shorter in length, had a higher degree of distinction due to faster interspecies variation, as well as a higher error. The 28S sequences were longer and had a certain degree of distinction between species (**Figure 6**).

**Figure 7:**
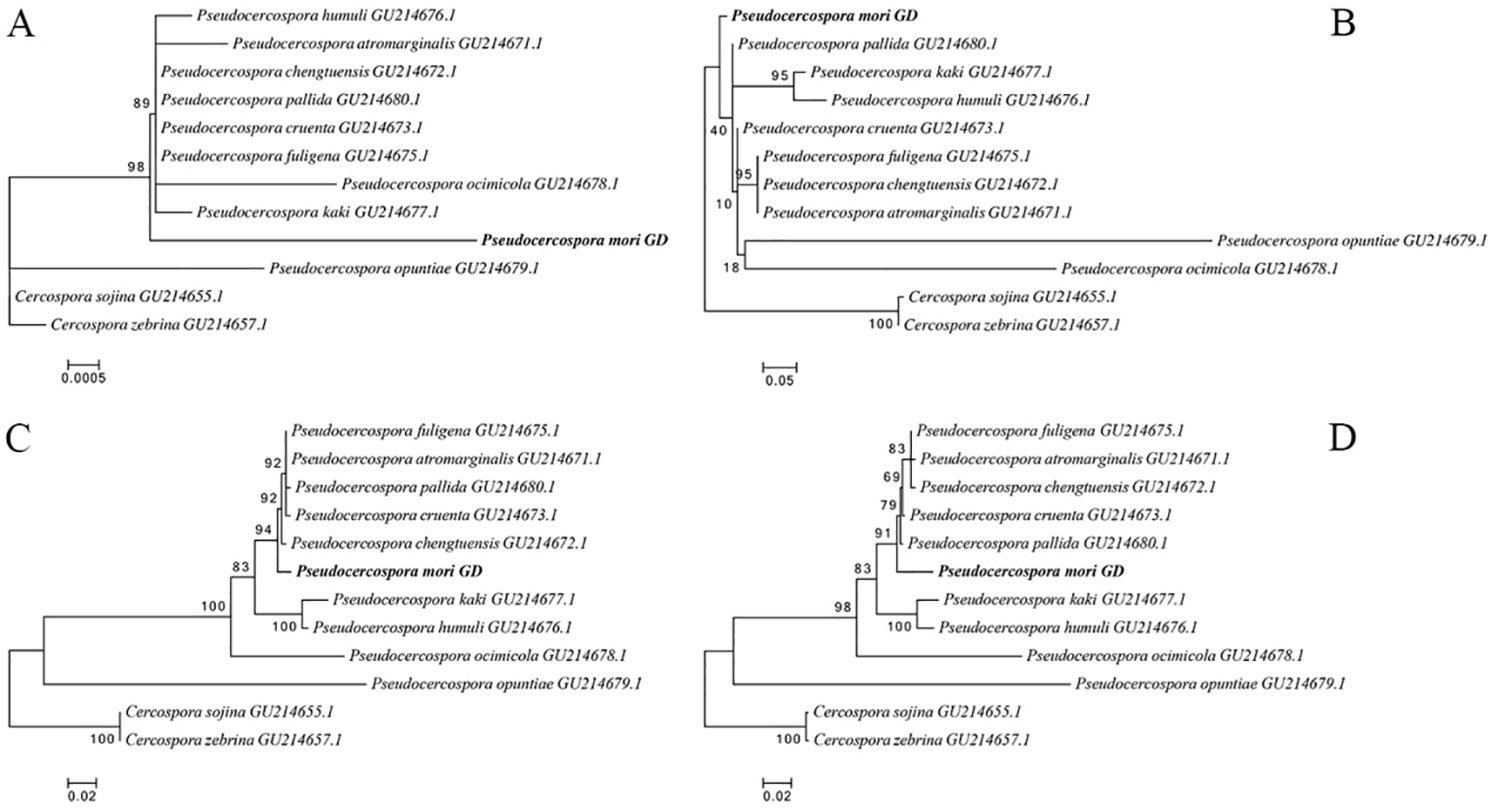
Phylogenetic trees constructed based on 18S **(A)**, ITS1 + 5.8S+ITS2 **(B)**, 28S **(C)**, and intact ribosomal DNA **(D)**, respectively. *Pseudocercospora mori* GD (PV770134) represents the sample from this study, while the remaining sequences are homologous fungal sequences retrieved from the NCBI database through BLAST comparison, with GenBank accession numbers provided in parentheses. Phylogenetic trees were constructed using the Maximum Likelihood (ML) method with 1,000 bootstrap replicates in MEGA 12.

The rDNA sequences were statistically analyzed by jModelTest2, and the alternative model GTR+G with the smallest AIC (Akaike Information Criterion) value was taken as the optimal phylogenetic tree construction model. After 1000 iterations using ML method, the evolutionary trees based on the 18S sequences (A), ITS1 + 5.8S+ITS2 sequences (B), 28S sequences (C), and intact rDNA (D) were calculated (**Figure 7**). The results showed that the 18S sequences were highly conserved with low interspecies differentiation. The ITS1 + 5.8S+ITS2 sequences had large errors, and the support for the branching structure of the tree was very low. The phylogenetic tree constructed using 28S sequence was closer to the intact rDNA phylogenetic tree in terms of structure, but it was not as accurate as the intact rDNA in terms of the calculation of genetic distances (**Figure 7**).

#### Application of high-throughput sequencing results

Based on the results of high-throughput sequencing, the genus *Pseudocercospora* was identified as the main fungal pathogen in the samples of mulberry leaf spot disease. Based on the rDNA and the full length of the mitochondrial genome, the NCBI Primer Blast Primer Premier software were used to design different primers required for downstream experiments (**Table 4**).

**Table 4:** PCR validation primers designed based on rDNA high-throughput sequencing and complete mitochondrial genome sequencing results.

Primer ID	Sequence (5’-3’)	Gene	Product size (bp)
W1724f	GCTACACTGACAGAGCCAACG	*rDNA*	473
W2196r	TGAAACTCCGACGCAAAGA		
A1956f	TCGGCAACGACCACCCA	*ITS*	730
A2685r	CTACCCAGAAGCATCCTCTACAAA		
1w7393F	CACTGCTTCTGCTTTCTTTT	*Cyt b*	490
1w7882R	TATTAAATAGACACCACTTC		
2w25369F	ATAACCGAGGTAGTCAGAGC	*COI*	700
2w26068R	CTTGTAGTGTATCTGTGTAA		

Complete mitochondrial genome of *Pseudocercospora mori* has been submitted to NCBI with accession number: MG543071.1.

W1724f/W2196r are primers for specific detection of *Pseudocercospora mori*; A1956f/A2685r are primers for amplification of fungal ITS region; 1H21668F/1H22222R, 2H13208F/2H13744R, 3H15185F/3H15896R, 5H5452F/5H6077R are primers for specific detection of *Pseudocercospora mori*; 1w7393F/1w7882R and 2w25369F/2w26068R are for mitochondrial *Cyt b* and *COI* genes partial segment amplification, respectively.

#### Validation of rDNA high-throughput sequencing results of the mulberry leaf spot disease pathogen

### Identification of mulberry leaf spot disease pathogens based on barcoded genes ITS, *Cyt b*, *COI*

**Figure 8:**
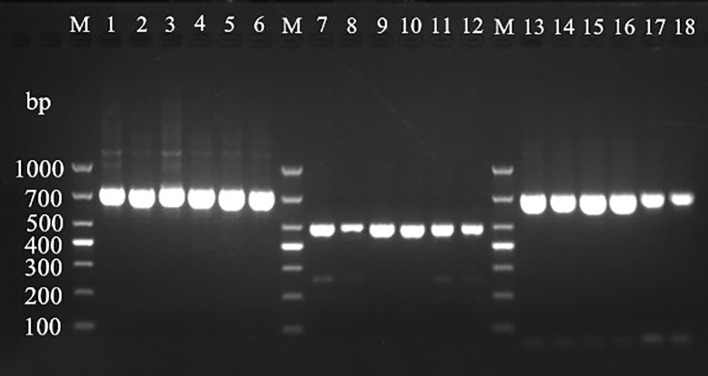
Electrophoretic gels of PCR amplified products of ITS, *Cyt b* and *CO Ι* genes of mulberry leaf spot disease pathogens in different regions. M: TaKaRa DL2000 Marker; lanes 1 to 6 are ITS region amplification primer set A1956f/A2685r; lanes 7 to 12 are *Cyt b* gene region amplification primer set 1w7393F/1w7882R; lanes 13 to 18 are *CO Ι* gene region amplification primer set 2w25369F/2w26068R; the DNA templates in lanes 1, 7 and 13 are diseased leaf spot samples from Guangzhou city; 2, 8 and 14 for diseased leaf spot samples from Wengyuan county, Shaoguan city, 3, 9 and 15 are diseased leaf spot samples from Yangshan county, Qingyuan city, 4, 10 and 16 for diseased leaf spot samples from Liannan Yao Autonomous county, Qingyuan city, 5, 11 and 17 for diseased leaf spot samples from Yingde city, Qingyuan city, 6, 12 and 18 for diseased leaf spot samples from Huazhou city, Maoming city.

Using the universal primers A1956f/A2685r of ITS region, mitochondrial *Cyt b*, and *COI* gene amplification primers, the DNA extracted from mulberry leaf spot disease samples was verified and identified. As shown in **Figure 8**, the results of PCR amplification of each primer group, lanes 1 to 6 (target fragment 730 bp), lanes 7-12 (target fragment 490 bp), and lane 3 (target fragment 700 bp) were obtained with target bands consistent with the expected results, indicating that the results were credible. The PCR products obtained by amplification using different primer sets were sent to Bioengineering for sequencing, and the sequencing results were subjected to phylogenetic analysis.

**Figure 9:**
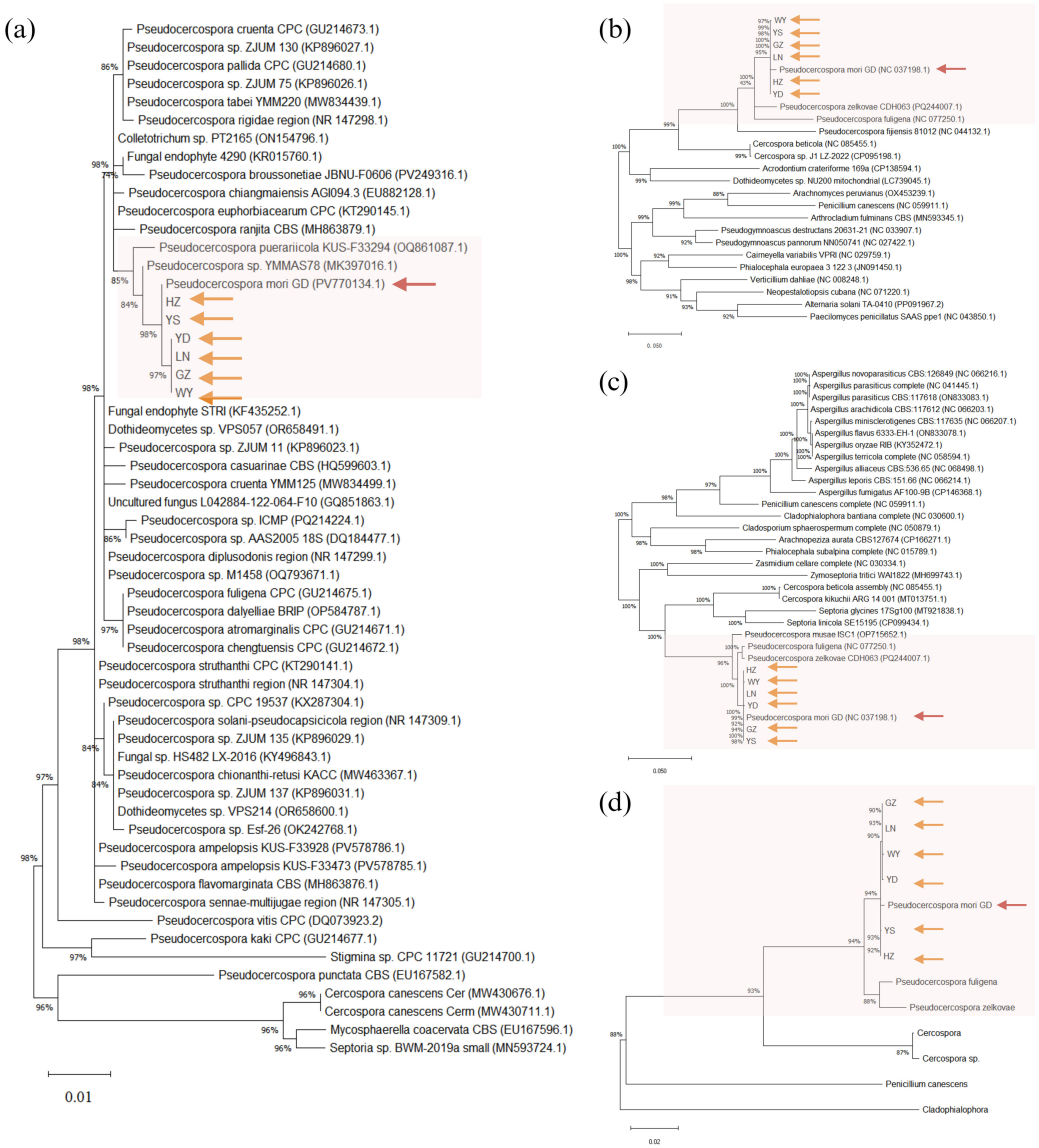
Phylogenetic trees constructed based on the sequences of ITS segment **(a)**, *COΙ* gene **(b)**, *Cyt b* gene **(c)**, and ITS-*Cyt b-COΙ* **(d)**. GZ, Guangzhou City, Guangdong Province sample; WY, Wengyuan County, Shaoguan City sample; YS, Yangshan sample; LN, Liannan Yao Autonomous County, Qingyuan City sample; YD, Yingde City, Qingyuan City sample; HZ, Huazhou City, Maoming City sample. Phylogenetic trees were constructed using the Maximum Likelihood (ML) method with 1,000 bootstrap replicates in MEGA12. The orange arrows represent the purified pathogen spores from the six samples (isolates) collected in this study. The red arrows represent the *Pseudocercospora mori* GD assembled by metagenomic sequencing in this study. The rest of the sequences are the sequences that were compared by the BLAST with the reported homologous sequences of the fungi in the NCBI database. The Genbank accession numbers of sequences are shown in parentheses. ITS-*Cyt b-COΙ* **(d)** reference gene accession numbers are detailed in **Table 5**.

As shown in **Figure 9**, the species with high homology and similarity to the sequencing results were all *Pseudocercospora*. There were no sequences in the NCBI database with 100% similarity to the present sequences, indicating that the present species might be an unreported species. Based on phylogenetic analysis of the ITS region (**Figure 9a**), *CO Ι* gene (**Figure 9b**), and *Cyt b* gene (**Figure 9c**), it was determined that this species belongs to *Pseudocercospora* genus. The identification was performed by submitting the ITS amplified sequence to the barcode database, and then identifying the species according to the barcode process. As shown in **Figure 9d**, most of the sequences in the ITS- *Cyt b*- *CO Ι* barcode database of *Pseudocercospora* were highly similar, but there was no complete overlap and no unique neighboring species sequences, indicating that the species could not be named according to the process. In addition, the molecular evolutionary tree constructed using ITS region (**Figure 9a**), *CO Ι* gene (**Figure 9b**), *Cyt b* gene (**Figure 9c**) and ITS- *Cyt b*- *CO Ι* (**Figure 9d**) of purified pathogenic spores collected from six locations in Guangdong province (i.e., GZ, WY, YS, LN, YD, and HZ) and the *Pseudocercospora mori* GD assembled by metagenomic sequencing clustered on the same branch, indicating that *Pseudocercospora mori* is the causal/epidemic pathogen of grey leaf spot disease of mulberry (*Morus atropurpurea*) in the present study.

**Table 5:** Reference sequences (with NCBI accession numbers) of ITS segment, *COI* gene, and *Cyt b* gene used to construct phylogenetic tree (**Figure 9d**).

Species	ITS	Cytb	COI
*Pseudocercospora mori*	PV770134.1	NC_037198.1	NC_037198.1
*Pseudocercospora fuligena*	GU214675.1	NC_077250.1	NC_077250.1
*Pseudocercospora zelkovae*	LC739836.1	PQ244007.1	PQ244007.1
*Cercospora beticola*	NR_121315.1	MW458938.1	NC_085455.1
*Cercospora* sp. J1 LZ-2022	OR122733.1	CP095198.1	CP095198.1
*Penicillium canescens*	NR_121256.1	NC_059911.1	NC_059911.1
*Cladophialophora bantiana*	PV138957.1	NC_030600.1	NC_030600.1

#### Results of re-inoculation experiments based on Koch’s postulates

**Figure 10:**
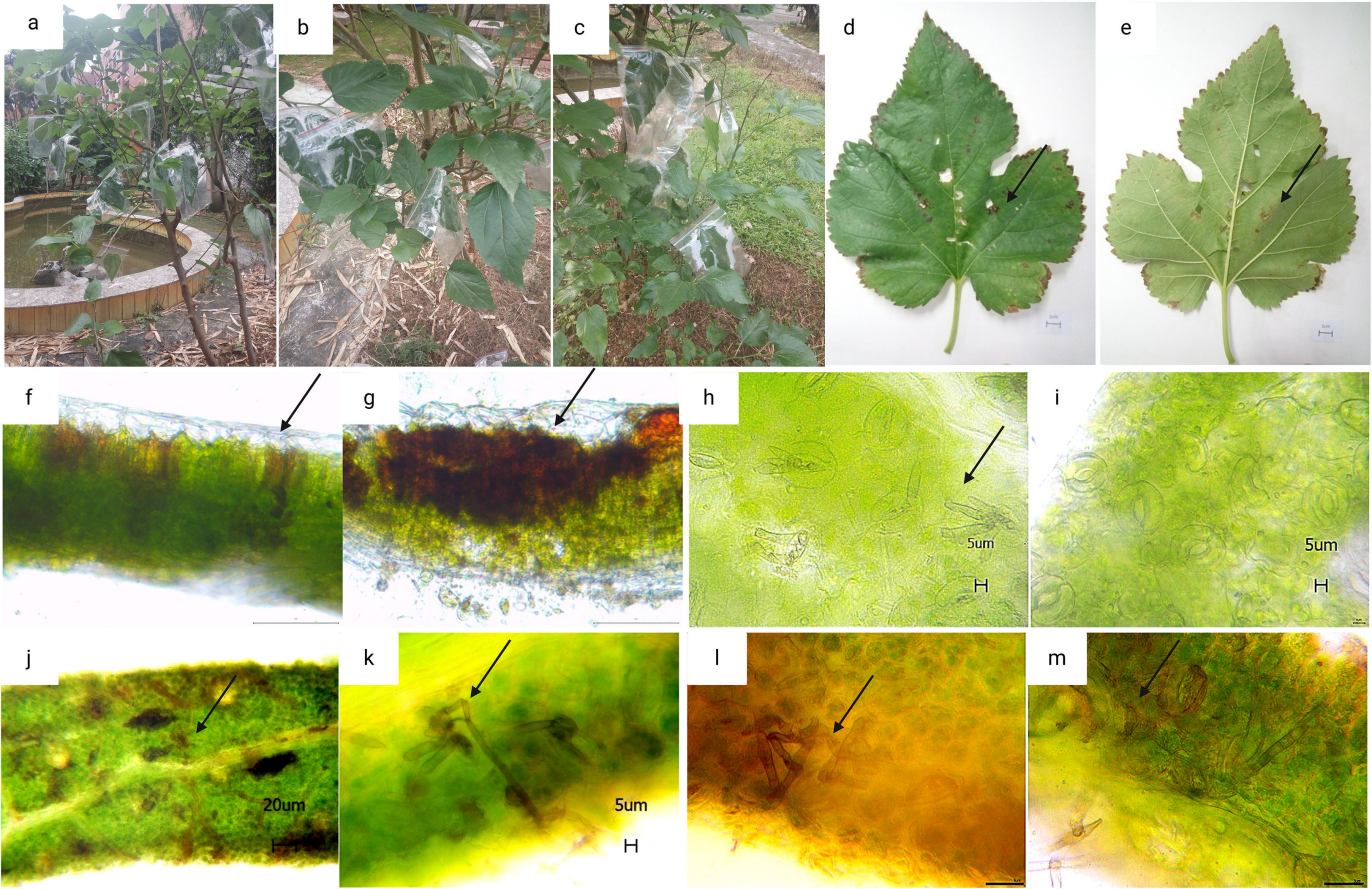
Re-inoculation experiment results based on the Koch’s postulates. **(a–c)** are three parallel samples of mulberry trees in the re-inoculation experiment. **(d–e)** mulberry leaves after 30 days of inoculation with pathogenic fungi (scale bar 1 cm); **(f, g)** tissue sections of a large and a small discolored spots (scale bar 100 μm); **(h, i)** leaf stomata and mycelia growth within the stomata after 30 days of inoculation with pathogenic fungi, where h is the stomata of inoculated mulberry leaves (inoculated group) and **(i)** is the stomata of healthy mulberry leaves (control group), the arrows point to the mycelia growth in the stomata (scale bar 5 μm).; **(j, k)** internal mycelia growth of the leaf after 30 days of inoculation with pathogenic fungi, the arrows point to the mycelia growth in the mesophyll tissue [scale bar 20 μm in **(j)** and 5 μm in **(k)**]; **(l, m)**: cross sections of leaf showing mycelia growth in the leaf mesophyll tissue, arrows indicate the mycelia growth in the leaf mesophyll tissue (scale bar is 20 μm).

After identifying the existence of the suspected causal pathogen, using Koch’s postulates, it was verified whether *Pseudocercospora mori* was the causal pathogen of mulberry leaf spot disease. It was determined whether it can infect leaves and cause leaf disease spot disease following re-inoculation. As shown in **Figure 10**, three healthy mulberry trees were selected for the validation of Koch’s postulates in this experiment. The Mulberry leaves infected with *Pseudocercospora mori* were the mature leaves of the middle and lower branches, with about 20 experimental leaves in each group (**Figures 10a–c**). In addition each mulberry tree was inoculated with about 10 leaves soaked with sterile water as a blank control group. Thirty days after inoculation, the corresponding typical symptoms appeared on leaves in all grafted samples (100%), and the diameter of the lesion varied from 0.5 to 1 cm. Discoloration spots (**Figures 10d, e**), i.e., necrosis of chloroplasts, were seen on the leaf surfaces. The discolored leaves were sectioned for further observation. It was observed that the discolored spots at the fenestrated tissue partly turned brown, indicating that the discolored spots on the leaf surface were caused by the necrosis and discoloration of the chloroplasts at the fenestrated tissue, however the spongy tissue was less affected (**Figures 10f**). A small brown discoloration was seen on the spongy tissue corresponding to the discolored fenestrated tissue (**Figures 10g**), and no mycelia (hyphae) growth was seen at the section, indicating that the existence of the discolored spots had no absolute relationship with the mycelium present on the back the leaf. Thirty days after inoculation, the infection entered the latent stage, and mycelia growth was observed within the stomata (**Figure 10h**). In contrast, the stomata of healthy mulberry leaves consisted of a pair of kidney-shaped defense cells held together and were seen to be free of any impurities (**Figure 10i**). Under the bright light, brown mycelium with a distinct color difference from the green leaf mesophyll tissue was visible (**Figure 10j**). After magnification, clear mycelium growing between the mesophyll cells was visible (**Figure 10k**). The observation of sectioned leaves in the latent phase of the re-inoculation experiment revealed that the free conidia were within the lower epidermis of the leaves (**Figure 10m**). Based on the direction of mycelium growth, it was seen that the mycelium gradually elongated into the spongy tissue from the lower epidermis (**Figure 10l**) and approached the fenestrated tissue (**Figure 10m**). No mycelium invasion into the fenestrated tissues was observed in the sections of several leaves examined in the re-inoculation experiment.

#### Detection of pathogenic fungi in soil and mulberry stems

### Validation of specific primers designed based on rDNA sequences

**Figure 11:**
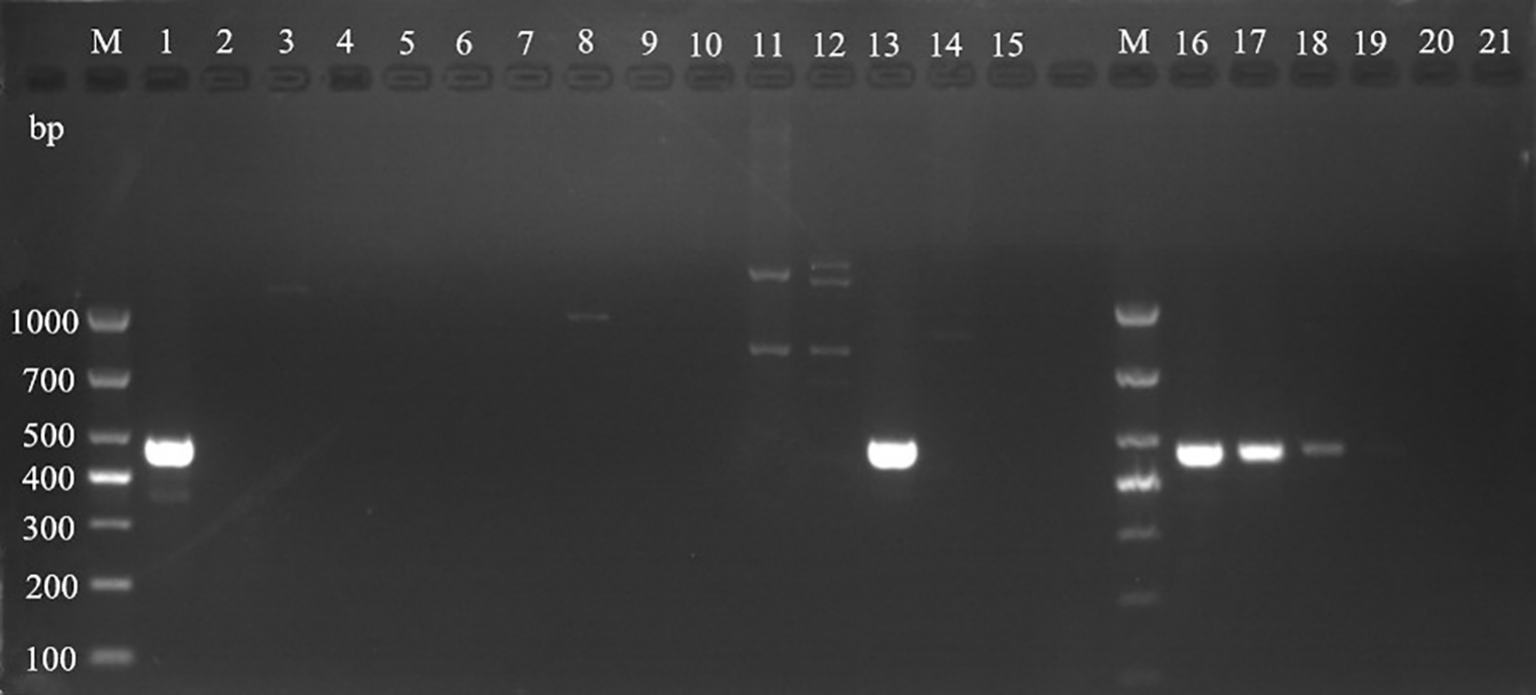
Validation of specific PCR primers designed based on *Pseudocercospora mori* rDNA. M: TaKaRa DL1000 Marker; lanes 1–15 are for primer (W1724f/W2196r) specificity test, and lanes 16–21 are for sensitivity test. The DNA templates for each lane are: 1: DNA of *Pseudocercospora mori*; 2: Cladosporium sp.; 3: *Penicillium verruculosum*; 4 *Aspergillus* sp.; 5: *Lecanicillium psalliotae*; 6: *Phanerina mellea*; 7: *Schizophyllum commune*; 8: *Candida mucifera*; 9: *Lasiodiplodia theobromae*; 10: *Pseudomonas aeruginosa*; 11: *Phyllactinia moricola*; 12: *Ciboria carunculoides*; 13: DNA of mulberry leaf spot disease pathogen (positive control); 14: DNA of mulberry tree (negative control); 15 water (blank control). Lanes 16–21 represent gradient dilutions of pathogen DNA. 16: 30 ng/μL; 17: 3 ng/μL; 18 is 3×10–^1^ ng/μL; 19: 3×10–^2^ ng/μL; 20: 3×10–^3^ ng/μL; and 21: 3×10–^4^ ng/μL.

Based on the full length rDNA sequences obtained through high-throughput sequencing, the specific primer W1724f/W2196 (**Table 4**) was designed using the NCBI Primer software. As shown in **Figure 11**, only templates of *Pseudocercospora mori* DNA in the positive control group (lane 1), and the mulberry leaf spot disease pathogen DNA of positive control (lane 13) produced bands at about 473 bp, while the other non-*Pseudocercospora mori* DNA templates had no bands or produced non-specific bands with a large difference in the size of the target band. The target fragment was brighter and had a single band, and there was no primer dimer (see lanes 1 and 13), indicating that the primer could be used for the specific detection of pathogen. The sensitivity of the primer was tested and acceptable results were obtained. The primers can detect the pathogen DNA diluted to 3×10–^2^ ng/μL (see lanes 16-19). Although the bands produced 3×10–^2^ ng/μL concentration was weak, it was still visible when magnified.

### Validation of presence of *Pseudocercospora mori* in the soil and on mulberry stems retrieved from mulberry orchards

**Figure 12:**
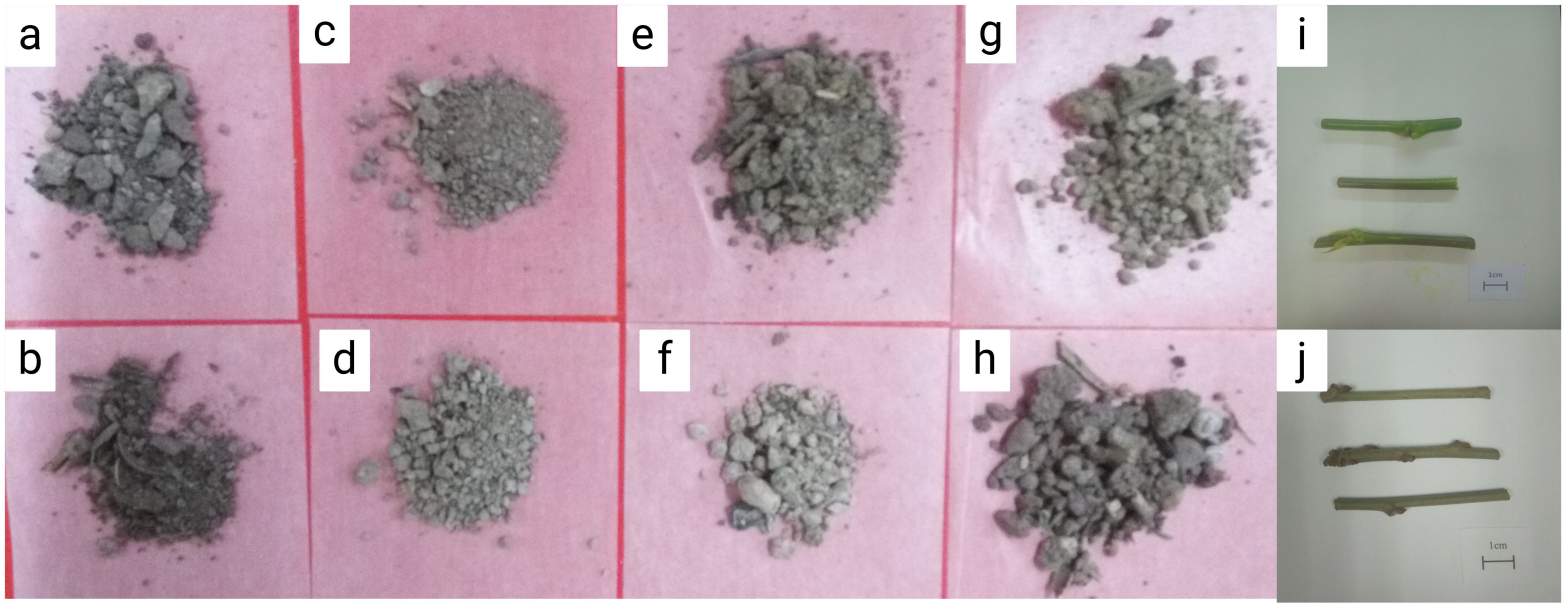
Mulberry fouling leaf disease related materials used to extract DNA of pathogenic bacteria. **(a)** soil of the laboratory mulberry planting area; **(b)**. soil samples collected from of the re-inoculation experimental area in this study; **(c, d)**. soil samples of the mulberry garden of Yingde Silkworm Breeding Farm, Yingde City; **(e–h)** soil samples collected from mulberry demonstration base of the South China Agricultural University; **(i)** mulberry twigs affected with leaf spot disease; **(j)** newly grown mulberry branches after winter pruning. Scale bar is 1 cm.

In this experiment, soil samples from eight mulberry growing areas were selected for DNA extraction. The samples were tested for the presence of suspected pathogenic fungi using *Pseudocercospora mori* specific detection primers t1724f/W2196r. As shown in **Figure 12a**, the DNA templates of the soil from the incidence area of Guangzhou city (**Figure 12a**: lane 1), Yingde city (**Figure 12a**: lanes 3-4), the mulberry demonstration base of the South China Agricultural University (**Figure 12a**: lanes 5-8), and the conidia-positive control of mulberry leaf spot disease pathogen (**Figure 12a**: lanes 9-10) produced bands of about 473 bp. These results indicated that the soil samples contained conidia of mulberry leaf spot disease pathogen. No bands were produced in the soil samples collected from of the re-inoculation experimental area (**Figure 12a**: lane 2), as well as in the negative control (**Figure 12a**: lanes 11-12).

Next, the twigs from the diseased mulberry (**Figures 12i, j**) were collected for DNA extraction and tested for the presence of the suspected pathogen using the specific detection primers W1724f/W2196r.

**Figure 13:**
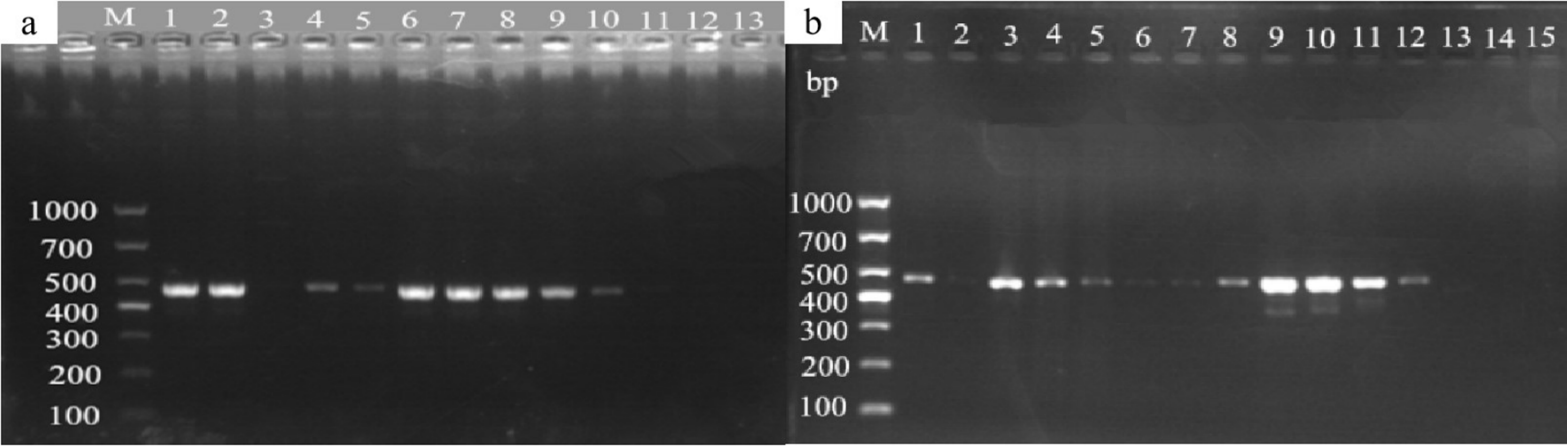
Detection of mulberry leaf spot disease pathogens in soil and twigs samples collected from different sources. **(a)** Mulberry leaf spot disease pathogen detection in soil from different sources. The DNA templates were as follows: 1. soil from the mulberry planting area in the laboratory; 2. soil samples collected from the re-inoculation experimental area; 3. soil from the mulberry garden of Yingde Silkworm Breeding Farm in Yingde City; 4. soil from the Yingde City; 5-8: soil samples from the demonstration base of the South China Agricultural University (taken from the four mulberry planting areas in the Mulberry Garden); Lanes 9-10. Mulberry leaf spot disease DNA (positive control); lane 11: Mulberry DNA (negative control); lane 13: sterile water (blank control). M TaKaRa DL5000 Marker; **(b)** Detection of the presence of pathogenic fungi in diseased mulberry trees. The DNA templates for each lane were extracted from the following materials: 1 and 3: diseased mulberry branches; 2: newly grown (non-diseased) mulberry branches; 4-5: mulberry leaves collected from re-inoculation experiment; 6. diseased mulberry leaves; 7–12 mulberry leaf spot disease pathogen DNA (positive control); 13–14 mulberry DNA (negative control); 15. sterile water (blank control); M TaKaRa DL5000 Marker.

As shown in **Figure 13b**, the specific bands were obtained at 473 bp for the diseased mulberry samples (13B, lanes 1, 3, 4, 5, and 6), as well as for the conidia-positive control of the mulberry leaf spot disease pathogen (13B, lanes 7-12). In contrast, no bands were obtained for the undiseased mulberry twigs (13B, lane 2), as well as for the negative control (**Figure 13b**: lanes 13-15), suggesting the presence of the suspected mulberry leaf spot pathogen in the mulberry samples.

## Discussion

**Figure 14:**
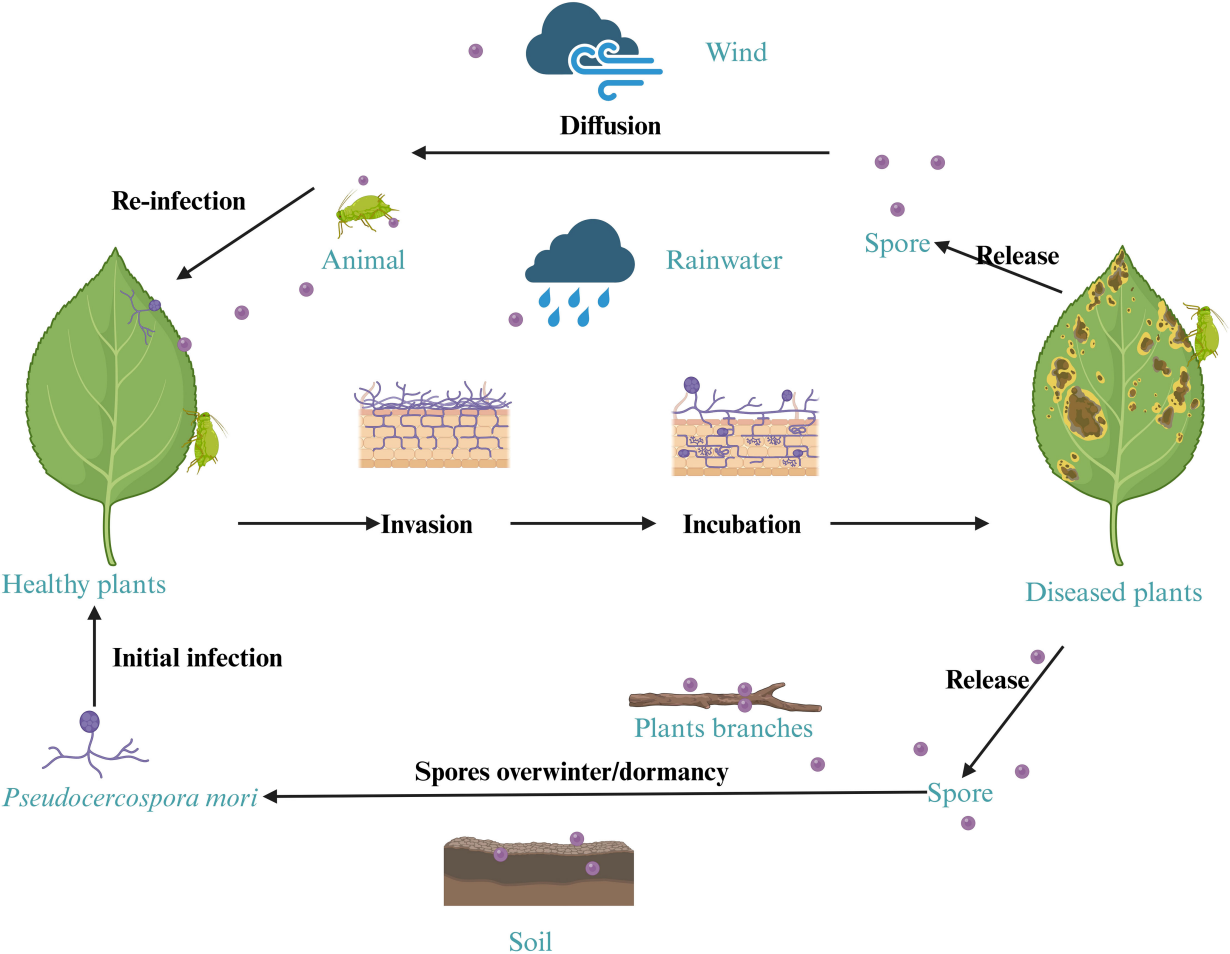
Simulated infection cycle of the mulberry leaf spot disease pathogen.

Mulberry grey leaf spot is an important disease that causes significant damages to mulberry production and sericulture industry (Kumar et al., 2011b). This study provides the first comprehensive characterization of grey leaf spot disease in mulberry caused by *Pseudocercospora mori* in multiple regions of Guangdong Province of China. The pathogen was consistently isolated from the diseased samples. We systematically investigated mulberry grey leaf spot disease through detailed observation of affected leaves, rigorous combination of morphological and molecular identification of the pathogen, isolation, and high-throughput sequencing. The pathogenicity of the suspected causal pathogen was confirmed through re-inoculation experiments, fulfilling the Koch’s postulates. This finding represents either a new geographic report or a previously undocumented association in this region, emphasizing the geographical variations in disease occurrence, pathogen diversity, the expanding distribution and potential impact of *Pseudocercospora mori* on mulberry cultivation. Our results substantially expand the known diversity of fungal pathogens causing leaf spot disease in *Morus* spp. and have important implications for disease diagnosis and management. *Pseudocercospora mori* has been previously identified as a causal pathogen of the grey leaf spot disease of mulberry in Japan, Democratic Republic of the Congo, the US, India, Pakistan, and Australia (Babu et al., 2002; Grice et al., 2006; Abbas et al., 2010; Niaz et al., 2010). Tellingly, the disease symptoms and pathogen characteristics vary depending on the disease onset period, mulberry variety, environmental conditions, and management practices. Although, *Pseudocercospora mori* has been previously documented in different parts of the world, our work makes a significant contribution to the phytopathological mapping of *Pseudocercospora mori*, particularly as its role has remained underreported in subtropical regions of China despite increasing foliar disease incidence in mulberry plantations. Based on the data obtained in our experiments, we have simulated the infection cycle of mulberry grey leaf spot pathogen in **Figure 14**.

### Symptoms of mulberry grey leaf spot disease

Mulberry grey leaf spot disease is characterized by diverse symptoms including grayish mold-like patches, black sooty spots, light gray areas, and powdery dark gray coatings (**Figure 2**) (Govindaiah et al., 2002; Maji et al., 2008). This variability stems not only from the characteristics of the pathogen *Pseudocercospora mori* but also from the interplay of climate, season, orchard management, mulberry variety, cultivation practices, and pest influences (Biswas et al., 1995; Chattopadhyay et al., 2011). The composition of foliar fungal communities significantly influences disease expression. For instance, Matić et al. (2019) found that traditionally saprophytic fungi, such as *Paramyrothecium foliicola*, can induce leaf spot diseases, suggesting that fungal ecological roles may shift with environmental conditions. Similarly, there may be mechanisms that regulate the different colors and causes of mulberry gray leaf spot disease symptoms.

Fungal community structure is shaped by host plant species and geographic location, which in turn influence disease manifestations. Darcy et al. (2020) demonstrated that leaf fungal communities in Hawaiian dicotyledonous plants correlate with host phylogeny and environmental factors (e.g., elevation), with geographic distance driving community divergence. Likewise, David et al. (2015) reported that aboveground fungal communities in the U.S. coastal dune ecosystems are constrained by host species and geographic barriers, while belowground communities are more influenced by environmental filtering. These findings suggest that regional variations in mulberry grey leaf spot symptoms may partly arise from differences in mulberry varieties and local fungal assemblages. For example, shrub-type and herbaceous mulberry differ in leaf surface microenvironments, affecting pathogen adhesion and infection efficiency (Govindaiah et al., 2002; Li et al., 2024).

Climate and season are pivotal drivers of symptom diversity. Optimal temperatures (20–30°C) and high humidity typically enhance fungal spore germination and infection (Zhao et al., 2022). However, extreme heat (>35°C) can impair spore viability, and excessive humidity (e.g., prolonged leaf wetness) may hinder mycelial growth by washing off spores or creating unfavorable conditions (Grice et al., 2006; Schoch et al., 2012). Effective orchard management, such as pruning and fertilization, significantly reduces disease severity, with well-maintained orchards exhibiting milder symptoms (Govindaiah et al., 2002; Rohela et al., 2025). Pests and concurrent diseases, like brown leaf spot, exacerbate symptom variability by compromising mulberry resistance (Biswas et al., 1995; Guha, 2025). This study observed no consistent correlation between leaf-back lesions and leaf-surface discoloration (**Figures 10d, e**), with discoloration linked to palisade cell necrosis (**Figures 10f, g**). While partially attributed to the pathogen, leaf aging and pest damage also contribute to disease progression (Biswas et al., 1995). Thus, the complexity of mulberry grey leaf spot symptoms reflects the synergistic effects of biotic and abiotic factors, necessitating an ecological approach for accurate diagnosis and management.

*Pseudocercospora* spp., including *Pseudocercospora mori*, have a broader ecological range and greater adaptability to diverse microclimatic conditions (Crous et al., 2013b; Nzioki et al., 2020). Importantly, mulberry leaf spot disease caused by *Phloeospora maculans* has been associated to high rainfall and humid environment in Korea, Turkey, and Nigeria (Soylu et al., 2003; Baiyewu et al., 2005; Hong et al., 2011). Previously, our group has reported mulberry zonate leaf spot disease, caused by fungal pathogen *Gonatophragmium mori*, occurring during the hot and humid months in Guangxi province of China (Luo et al., 2024). Similarly, *Pseudocercospora fijiensis* in banana and *Pseudocercospora musae* in plantain have been reported to have an affinity with humid microenvironments (Arzanlou et al., 2008; Churchill, 2011; Crous et al., 2013b). This adaptability of the related fungal pathogens may explain the prevalence and widespread distribution of *Pseudocercospora mori* observed in different localities surveyed in this study.

### Morphological and molecular identification of the pathogen

In the present study, the isolated pathogen causing mulberry grey leaf spot exhibited characteristic *Pseudocercospora* morphology, including internal hyphae, external hyphae, stromata and clavate conidia (**Figure 3**). *Pseudocercospora*, belonging to the Mycosphaerellaceae (Ascomycota), typically produces dark, elongated, multi-septate conidia with simple stromatic structures and hyphae that often invade hosts via stomata (Crous et al., 2013a; Dobbs et al., 2024). Through morphological analysis and host specificity, this study excluded related genera such as *Clasterosporium*, *Sirosporium*, and *Cercospora*, initially identifying the pathogen as *Pseudocercospora mori* (Liu and Guo, 1998). This aligns with reports of mulberry gray leaf spot caused by *Pseudocercospora mori* in Australia (Grice et al., 2006), Iran (Hesami et al., 2012), and Pakistan (Niaz et al., 2010), indicating its global occurrence in mulberry (*Morus* spp.).

The infection process further reveals the pathogen’s morphology. *Pseudocercospora mori* produces primary infection hyphae, internal hyphae, and secondary infection hyphae (Babu et al., 2002). Primary hyphae, derived from conidia, penetrate leaves directly or via stomatopodia, which swell into vesicle-like structures in stomatal chambers. Internal hyphae form compact stromata in substomatal cavities, each bearing 1–5 conidia on short, unbranched conidiophores. Secondary hyphae, arising from internal hyphae or stromata, emerge onto the leaf surface, branching extensively and producing single conidia morphologically similar to stromatic conidia. Conidia are obclavate to cylindrical, with 3–17 cells, measuring 4–35 × 1.5–2 μm (Babu et al., 2002). In the present study, we recorded elongated conidia (33.779–68.286 × 3.638–5.649 μm, 6 septa) and short conidia (20.368–36.605 × 3.560–5.310 μm, 3 septa), partially overlapping with report of Babu et al., 2002, with septa number and aspect ratio varying by environment (Guo and Liu, 2005). Calcofluor White and Fluorescent Brightener 28 staining confirmed chitin in conidial walls (**Figures 4a–d**), while AOPI staining showed that some conidia remain viable (**Figures 4e, f**), suggesting prolonged survival and dispersal under favorable conditions (Canto-Canché et al., 2023).

Although morphological identification methods are indispensable for preliminary assessments, they often fall short when dealing with morphologically complex and underreported fungal taxa. Morphological identification relies on phenotypes, whereas molecular approaches reveal interspecies differences through DNA sequences (Raja et al., 2017; Zaman et al., 2025). Therefore, for confirming taxonomic placement of the causal pathogen with high resolution, the present study employed molecular identification through high-throughput sequencing and validated the pathogen identity, with *Pseudocercospora* sequences showing the highest abundance (25.10%, **Table 2**). The rDNA sequence (5469 bp, GC content 50.67%) included 18S, ITS1, 5.8S, ITS2, and 28S regions, with ITS lacking a 100% match, suggesting potential species-level variation (Romanelli et al., 2014). Additionally, *Cyt b* and *COI* sequences indicated close affinity to *Pseudocercospora*, but limited *COI* database coverage hindered species-level resolution (Crous et al., 2013a). The 18S region was highly conserved, ITS showed high variability with increased error rates, and 28S was more reliable (**Supplementary Figures 2** and **3**). Combining high-throughput sequencing and host traits, the pathogen was confirmed as *Pseudocercospora mori* (**Figure 9**). This study used ITS, *Cyt b*, and *COI* to confirm genus-level classification, but ITS conservation and database limitations necessitate additional genes like *EF-1α* for species-level resolution (Crous et al., 2013a; González-Sayer et al., 2022).

Initially, we employed PDA as the basic medium to isolate the target fungal pathogen from diseased mulberry leaves exhibiting leaf spot symptoms, however, artificial culturing of *Pseudocercospora mori* proved challenging, likely due to its specific growth requirements (Groenewald et al., 2024; de Jesus et al., 2025). We were successful in isolating a number of other filamentous fungi (data not shown). None of those were confirmed as the primary mulberry leaf spot pathogen based on pathogenicity tests and symptom reproduction, indicating that PDA medium was unsuitable for isolating the target (*Pseudocercospora mori*) pathogen. The successful purification of viable spores via host-based inoculation on fresh mulberry leaves suggests that the pathogen has host-specific growth requirements rather than inherent unculturability.

High-throughput sequencing overcame above limitations, verifying the pathogen’s presence (Li et al., 2021). Genomic studies of other *Pseudocercospora* species, such as *Pseudocercospora macadamiae* and *Pseudocercospora ceratoniae*, have elucidated genetic diversity and pathogenicity mechanisms, guiding future molecular classification of *Pseudocercospora mori* (Akinsanmi and Carvalhais, 2020; Perrotta et al., 1998). Integrating morphology and molecular data is critical for confirming *Pseudocercospora mori*, though environmental impacts on conidial morphology warrant further investigation to clarify taxonomic significance as argued for other pathogenic fungi. Additionally, the specific impacts of environmental factors, such as temperature and humidity on conidial germination, remain underexplored. Future research should focus on developing portable diagnostic tools for rapid detection, integrating multi-gene sequencing (e.g., *EF-1α*, *RPB2*) to refine taxonomic classification, and investigating the molecular interactions between *Pseudocercospora mori* and mulberry hosts to better understand pathogenesis and inform control strategies.

### Pathogen detection and dissemination

Given the limitations of morphological identification and the cryptic nature of symptom expression in early infection stages, we developed a PCR-based detection method specific for *Pseudocercospora mori*. The PCR detection with *rDNA* sequence of *Pseudocercospora mori* W1724f/W2196r primers was sensitive and specific, as no cross-reactivity was observed with DNA extracted from other pathogens, including related fungal species. Given the long procedural time of culture-based isolation methods, which require 5–7 days for fungal isolation and sporulation, PCR-based detection offers a faster turnaround time, enhancing the feasibility of field diagnostics. Importantly, in the present study, PCR with W1724f/W2196r primers enabled detection of *Pseudocercospora mori* during the early phase. This is particularly critical for narrowing the control window and the timely implementation of disease management measures (Li et al., 2021). From a practical perspective, the whole genome sequences obtained and assembled, along with the primer set (W1724f/W2196r) developed and validated in this study, could be utilized to develop quantitative PCR (qPCR) and field-friendly LAMP assays, thereby enhancing the scalability and practicality of *Pseudocercospora mori* monitoring, especially in large-scale mulberry production systems.

Soil serves as a critical reservoir for microbes and plant debris, facilitating their survival and spread. Like other fungal pathogen, *Pseudocercospora mori* remains viable in surrounding soil and fallen mulberry tissues. This study employed fungus-specific primers to extract DNA from mulberry orchard soil (**Figures 12a–h**), optimizing the protocol to minimize OD measurement errors and ensure reliable detection. The DNA concentration of *Pseudocercospora mori* in soil ranged from 3×10⁻³ to 3×10⁻¹ ng/μL, corresponding to an estimated spore density of 10²–10³ spores/g. This suggests that 10^6^–10^7^ spores may accumulate around each mulberry tree, far exceeding the threshold for disease outbreaks (Crous et al., 2023). These findings highlight soil as a primary dissemination source for *Pseudocercospora mori*, particularly in high-density orchards (Biswas et al., 1995). AOPI staining revealed that spores retained viability under cold storage (**Figures 4e, f**), and the mild, humid climate of Guangdong (20–30°C, high humidity) further prolongs spore survival, amplifying dissemination risks (Furuya et al., 2025). Infected leaves and twig debris contribute to persistent disease cycles by dispersing spores via rain splash or wind (Grice et al., 2006; Dofuor et al., 2024). Based on this evidence, it is recommended that, in addition to removing diseased leaves, winter field management in mulberry orchards should include disinfection and sterilization of both soil and twig surfaces to effectively prevent the occurrence of grey leaf spot disease.

The Koch’s postulates confirmed the pathogenicity of *Pseudocercospora mori* (**Figure 10**). Thirty days post-inoculation, leaves exhibited characteristic discoloration spots (**Figures 10d, e**), with hyphae penetrating spongy tissue via stomata but not reaching palisade tissue, indicating a preference for superficial and mid-layer infection (Babu et al., 2002; Alves et al., 2021). The DNA detection verified the presence of *Pseudocercospora mori* nucleic acids in diseased leaves (**Figure 13b**), soil (**Figure 13a**), and twigs (**Figure 13b**), confirming its persistence beyond leaves in soil residues and woody debris (Govindaiah et al., 2002). Twigs, as potential source of infection, may spread the pathogen through pruning or transport, warranting special attention (Wang, 2009). This multi-pathway survival complicates disease management, particularly in continuous cropping systems.

Although *Pseudocercospora mori* rarely infects beyond the Moraceae family, cross-infection risks among Moraceae plants (e.g., *Morus* and *Ficus*) remain a concern (Hesami et al., 2012). Avoiding cultivation of other Moraceae crops near mulberry orchards is recommended to limit host availability. Future research should investigate antagonistic interactions between soil microbial communities and *Pseudocercospora mori*, alongside quantifying the effects of rainfall and soil moisture on spore dispersal (Darcy et al., 2020; Zhao et al., 2022). Integrating molecular diagnostics, ecological management, and sustainable control measures will pave the way for effective, long-term management of mulberry grey leaf spot disease in the Guangdong region.

## Conclusion

In this study, traditional morphological methods, high-throughput sequencing, molecular phylogenetic analysis, and pathogenicity tests were used to identify the main causal pathogen of grey leaf spot disease in mulberry samples collected from different localities of Guangdong province of China. We obtained the full sequences of rDNA and mitochondrial genome of *Pseudocercospora* spp. Based on the sequencing results, primers sets were designed for PCR amplification and sequencing of ITS, *Cyt b*, and *CO I* gene segment sequences, and phylogenetic analysis. The molecular phylogenetic results showed that the pathogenic fungi belonged to the family Mycosphaerellaceae and genus *Pseudocercospora*. Combing morphological features and molecular evidence and the host, *Pseudocercospora mori* was identified as a main causal pathogen of mulberry leaf spot disease. PCR primers specifically designed based on the rDNA sequence of *Pseudocercospora mori* achieved a detection sensitivity as low as 3 × 10⁻² ng/μL. This assay enables early-stage diagnosis from infected leaf tissue and is also applicable for pathogen detection on the surface of mulberry twigs and in orchard soil. These results substantially expand the known diversity of fungal pathogens associated with leaf spot disease in *Morus* spp., with important implications for disease diagnosis and management at the field level. Future studies should focus on elucidating the mechanisms of infection and host–pathogen interactions.

## Data Availability

All data generated or analyzed during this study are included in this article and its supplementary information files. Metagenomic data have been submitted to NCBI with the accession numbers: PRJNA1272820, SAMN48924798, and SRR33885040. *Pseudocercospora mori* GD mitochondria complete genome have been submitted to NCBI with the accession number: NC_037198.1. *Pseudocercospora mori* GD nuclear rRNA/ITS complete genome have been submitted to the NCBI with the accession number: PV770134. *COI* (GenBank PV780452-PV780457); *Cytb* (GenBank PV806600-PV806605); ITS (GenBank PX062118-PX062123).

## References

[B1] Abbas S. Q. Ali I. Niaz M. Ayesha R. Iftikhar T. (2010). New fungal records on Morus alba from Faisalabad Pakistan I. Pak. J. Bot. 42, 583–592.

[B2] Agrios G. N. (2005). Plant Pathology. 5th ed (United States of America: Academic Press, Elsevier).

[B3] Akinsanmi O. A. Carvalhais L. C. (2020). Draft genome of the macadamia husk spot pathogen, *Pseudocercospora macadamiae* . Phytopathology 110, 1623–1629. doi: 10.1094/PHYTO-12-19-0460-A, PMID: 32343617

[B4] Alves R. F. Marques J. P. R. Appezzato-da-Glória B. Spósito M. B. (2021). Process of infection and colonization of *Pseudocercospora kaki* in persimmon leaves. J. Phytopathol. 169, 168–175. doi: 10.1111/jph.12971

[B5] Ávila A. Groenewald J. Z. Trapero A. Crous P. W. (2005). Characterisation and epitypification of *Pseudocercospora cladosporioides*, the causal organism of *Cercospora* leaf spot of olives. Mycological Res. 109, 881–888. doi: 10.1017/S0953756205003503, PMID: 16175790

[B6] Arunakumar G. S. NR N. P. M. Arya N. R. Monika B. M. Sherpa D. C. Anupama C.. (2023). Diversity of fungal pathogens in leaf spot disease of Indian mulberry and its management. Heliyon 9, e21750. doi: 10.1016/j.heliyon.2023.e21750, PMID: 38027777 PMC10665727

[B7] Arzanlou M. Groenewald J. Z. Fullerton R. A. Abeln E. C. Carlier J. Zapater M. F.. (2008). Multiple gene genealogies and phenotypic characters differentiate several novel species of Mycosphaerella and related anamorphs on banana. Persoonia-Molecular Phylogeny Evol. Fungi 20, 19–37. doi: 10.3767/003158508X302212, PMID: 20467484 PMC2865351

[B8] Babu A. M. Kumar V. Govindaiah. (2002). Surface ultrastructural studies on the infection process of *Pseudocercospora mori* causing grey leaf spot disease in mulberry. Mycological Res. 106, 938–945. doi: 10.1017/S095375620200624X

[B9] Bai X. Zhao X. Liu K. Yang X. He Q. Gao Y.. (2024). Mulberry leaf compounds and gut microbiota in Alzheimer’s disease and diabetes: A study using network pharmacology, molecular dynamics simulation, and cellular assays. Int. J. Mol. Sci. 25, 4062. doi: 10.3390/ijms25074062, PMID: 38612872 PMC11012793

[B10] Baiyewu R. A. Amusa N. A. Idowu J. B. (2005). Leaf spot in mulberry plant (Morus alba) in the lowland humid tropics of southwestern Nigeria. Plant Pathol. J. 4 (2), 103-106. doi: 10.3923/ppj.2005.103.106

[B11] Biswas S. Das N. K. Qadri S. M. H. Saratchandra B. (1995). Evaluating different plant extracts against three major diseases of mulberry. Indian Phytopathol. 48, 342–346.

[B12] Canto-Canché B. Burgos-Canul Y. Y. Chi-Chuc D. Tzec-Simá M. Ku-González A. Brito-Argáez L.. (2023). Moonlight-like proteins are actually cell wall components in *Pseudocercospora Fijiensis* . World J. Microbiol. Biotechnol. 39, 232. doi: 10.1007/s11274-023-03676-3, PMID: 37349471

[B13] Chan E. W. Lye P. Y. Wong S. K. (2016). Phytochemistry, pharmacology, and clinical trials of Morus alba. Chin. J. Natural Med. 14, 17–30. doi: 10.3724/SP.J.1009.2016.00017, PMID: 26850343

[B14] Chattopadhyay S. Ali K. A. Doss S. G. Das N. K. Aggarwal R. K. Bandopadhyay T. K.. (2011). Association of leaf micro-morphological characters with powdery mildew resistance in field-grown mulberry (*Morus* spp.) germplasm. AoB Plants 2011, plr002. doi: 10.1093/aobpla/plr002, PMID: 22476473 PMC3244759

[B15] Choi Y. W. Hyde K. D. Ho W. H. (1999). Single spore isolation of fungi. Fungal Diversity. 3, 29-38.

[B16] Churchill A. C. L. (2011). Mycosphaerella Fijiensis, the black leaf streak pathogen of banana: Progress toward understanding pathogen biology and detection, disease development, and the challenges of control. Mol. Plant Pathol. 12, 307–328. doi: 10.1111/j.1364-3703.2010.00672.x, PMID: 21453427 PMC6640443

[B17] Crous P. W. Braun U. Hunter G. C. Wingfield M. J. Verkley G. J.M. Shin H. D.. (2013a). Phylogenetic lineages in *pseudocercospora* . Stud. Mycology 75, 37–114. doi: 10.3114/sim0005, PMID: 24014898 PMC3713886

[B18] Crous P. W. Braun U. Hunter G. C. Wingfield M. J. Verkley G. J. M. Groenewald J. Z. (2013b). Phylogenetic lineages in pseudocercospora. Stud. Mycology 75, 1–49. doi: 10.3114/sim0015, PMID: 24014898 PMC3713886

[B19] Crous P. W. Osieck E. R. Shivas R. G. Tan Y. P. Bishop-Hurley S. L. Esteve-Raventós F.. (2023). Fungal Planet description sheets: 1478–1549. Persoonia-Molecular Phylogeny Evol. Fungi 50, 158–310. doi: 10.3767/persoonia.2023.50.05, PMID: 38567263 PMC10983837

[B20] da Costa C. A. Veloso J. S. de Oliveira B. F. Lourenco V. Jr. Reis A. (2021). First report of Neophloeospora maculans causing leaf spots in Morus nigra and M. alba in Brazil. J. Plant Dis. Prot. 128, 317–321. doi: 10.1007/s41348-020-00370-6

[B21] Darcy J. L. Swift S. O. I. Cobian G. M. Zahn G. L. Perry B. A. Amend A. S. (2020). Fungal communities living within leaves of native Hawaiian dicots are structured by landscape-scale variables as well as by host plants. Mol. Ecol. 29, 865–878. doi: 10.1111/mec.15544, PMID: 32640084

[B22] David A. S. Seabloom E. W. May G. (2015). Plant host species and geographic distance affect the structure of aboveground fungal symbiont communities, and environmental filtering affects belowground communities in a coastal dune ecosystem. Microbial Ecol. 71, 912–925. doi: 10.1007/s00248-015-0712-6, PMID: 26626912

[B23] de Jesus J. A. Monteiro S. L. C. Bragança C. A. D. Rocabado J. M.A. D’Ávila L. S. da Invencao D. R.S.. (2025). *Pseudocercospora Fijiensis* and *Pseudocercospora musae*: Understanding the relationship between biology and epidemiology. Fungal Biol. Rev. 51, 100408. doi: 10.1016/j.fbr.2024.100408

[B24] Deng Y. Li C. Chen Y. Zou Z. Gong J. Shen C.. (2024). Chemical profile and aroma effects of major volatile compounds in new mulberry leaf fu brick tea and traditional fu brick tea. Foods 13, 1808. doi: 10.3390/foods13121808, PMID: 38928750 PMC11203251

[B25] Dobbs J. T. Caballero J. R. I. Ata J. P. Babiker E. Copes W. E. Stewart J. E.. (2024). Genomic and transcriptomic comparisons of the twig blight pathogen, *Passalora sequoiae*, with Mycosphaerellaceae foliar and conifer pathogens. Phytopathology 114, 732–742. doi: 10.1094/PHYTO-08-23-0271-R, PMID: 37942864

[B26] Dofuor A. K. Obeng J. Sossah F. L. Osabutey A. F. Lutuf H. Osei‐Owusu J.. (2024). A comprehensive review of *Pseudocercospora* fruit and leaf spot (angular leaf spot): Current status, advances and future directions for sustainable citrus production. Plant Pathol. 73, 1708–1718. doi: 10.1111/ppa.13947

[B27] Furuya H. Shimizu S. Kamijima H. (2025). Influence of daily temperature and relative humidity durations on lesion development of black leaf mold (*Pseudocercospora fuligena*) in greenhouse-grown tomato. PhytoFrontiers 4, 123–132. doi: 10.1094/PHYTOFR-02-24-0007-R

[B28] Gao R. Zhang G. (2013). Potential of DNA barcoding for detecting quarantine fungi. Phytopathology. 103 (11), 1103–1107. doi: 10.1094/PHYTO-12-12-0321-R, PMID: 23718836

[B29] González-Sayer S. Oggenfuss U. García I. Aristizabal F. Croll D. Riaño-Pachon D. M. (2022). High-quality genome assembly of *Pseudocercospora ulei*, the main threat to natural rubber trees. Genet. Mol. Biol. 45, e50510051. doi: 10.1590/1678-4685-GMB-2021-0051, PMID: 35037932 PMC8762716

[B30] Govindaiah G. Naik V. N. Sharma D. D. Gupta V. P. (2002). Occurrence of grey leaf spot disease caused by *Pseudocercospora mori* (Hara) Deighton, affecting mulberry in South India. Indian J. Sericulture 41, 64–65.

[B31] Guo Y. Liu X. (2005). Chinese Fungi. Vol. 24: Cercospora (Beijing: Science Press), pp. 195–327.

[B32] Grice K. R. E. Beilharz V. Shivas R. G. (2006). First record of *Pseudocercospora mori* causing grey leaf spot on mulberry in Australia. Australas. Plant Dis. Notes 1, 9–10. doi: 10.1071/DN06005

[B33] Groenewald J. Z. Chen Y. Y. Zhang Y. Roux J. Shin H. D. Shivas R. G.. (2024). Species diversity in *pseudocercospora* . Fungal Systematics Evol. 13, 29. doi: 10.3114/fuse.2024.13.03, PMID: 39135885 PMC11317867

[B34] Guha A. (2025). Common Diseases of Field Crops and Their Management. Educohack Press.

[B35] Guo Yinglan Liu Xijin (2005). Chinese Fungi (Volume 24): Cercospora. Beijing: Science Press, 195–327. (in Chinese)

[B36] Guo L. Shi X. Cao F. Hu S. Qian W. (2025). Effects of dietary addition of mulberry leaf powder on blood metabolites and fecal microbiota composition in Hu sheep. Front. Anim. Sci. 5, 1469850. doi: 10.3389/fanim.2024.1469850

[B37] Hawksworth D. L. (2003). Mycological research news. Mycological Res. 107, 1249–1250. doi: 10.1017/S0953756203219158

[B38] Hebert P. D. N. Ratnasingham S. de Waard J. R. (2003). Barcoding animal life: Cytochrome c oxidase subunit 1 divergences among closely related species. Proc. R. Soc. B 270, 96–99. doi: 10.1098/rsbl.2003.0025, PMID: 12952648 PMC1698023

[B39] Hesami S. Khodaparast S. A. Zare R. (2012). New reports on *Cercospora* and *Pseudocercospora* from Guilan province (N Iran). Rostaniha 13, 95–100. doi: 10.22092/botany.2012.101391

[B40] Hong S. K. Kim W. G. Sung G. B. Choi H. W. Lee Y. K. Shim H. S.. (2011). Occurrence of leaf spot on mulberry caused by Phloeospora maculans in Korea. Plant Pathol. J. 27, 193–193. doi: 10.5423/PPJ.2011.27.2.193

[B41] Hou J. Ji X. Chu X. Wang B. Sun K. Wei H.. (2024). Mulberry leaf dietary supplementation can improve the lipo-nutritional quality of pork and regulate gut microbiota in pigs: A comprehensive multi-omics analysis. Animals 14, 1233. doi: 10.3390/ani14081233, PMID: 38672381 PMC11047539

[B42] Hu T. Yu Y. Wu J. Xu Y. Xiao G. An K.. (2024). Structural elucidation of mulberry leaf oligosaccharide and its selective promotion of gut microbiota to alleviate type 2 diabetes mellitus. Food Sci. Hum. Wellness 13, 2161–2173. doi: 10.26599/FSHW.2022.9250180

[B43] Huang X. Yuan T. Huang Y. Qazi I. H. Liu J. (2025). Analysis of causal pathogens of mulberry bacterial blight in samples collected from eight provinces of China using culturomics and metagenomic sequencing methods. Front. Plant Sci. 16, 1517050. doi: 10.3389/fpls.2025.1517050, PMID: 40093613 PMC11906434

[B44] Huang Z. Y. Zhu X. K. (2013). Investigation and control of mulberry leaf blight in Nanyao Village, Hanyin County. Northern Sericulture 34, 27–28.

[B45] Ji X. Lu G. Gai Y. Zheng C. Mu Z. (2008). Biological control against bacterial wilt and colonization of mulberry by an endophytic Bacillus subtilis strain. FEMS Microbiol. Ecol. 65, 565–573. doi: 10.1111/j.1574-6941.2008.00543.x, PMID: 18631174

[B46] Kumar P. M. P. Qadri S. M. H. Pal S. C. (2011b). Factors influencing development and severity of grey leaf spot of mulberry (Morus spp.). Int. J. Ind. Entomology Biomaterials 22, 11–15. doi: 10.7852/ijie.2011.22.1.11

[B47] Kumar P. M. P. Qadri S. M. H. Pal S. C. Pal S. C. Misra A. K. (2011a). Quantification of relation between disease intensities and physiological and biochemical changes in mulberry due to grey leaf spot. Indian J. Sericulture 50, 28–33.

[B48] Kumar S. Stecher G. Suleski M. Sanderford M. Sharma S. Tamura K. (2024). MEGA12: Molecular Evolutionary Genetic Analysis version 12 for adaptive and green computing. Mol. Biol. Evol. 41, msae263. doi: 10.1093/molbev/msae263, PMID: 39708372 PMC11683415

[B49] Li L. Ahmed S. Abdulraheem M. I. Hussain F. Zhang H. Wu J.. (2024). Plant microbe interaction—Predicting the pathogen internalization through stomata using computational neural network modeling. Foods 13, 3848. doi: 10.3390/foods13233848, PMID: 39682918 PMC11640189

[B50] Li N. Cai Q. Miao Q. Song Z. Fang Y. Hu B.. (2021). High-throughput metagenomics for identification of pathogens in the clinical settings. Small Methods 5, 2000792. doi: 10.1002/smtd.202000792, PMID: 33614906 PMC7883231

[B51] Liao S. T. Xiao G. S. (2013). Mulberry: A plant with homologous origins of medicine, food, and feed (Beijing: China Agricultural Science and Technology Press), 4–16.

[B52] Lin Z. X. Wang C. J. Tu H. W. Tsai M. T. Yu M. H. Huang H. P. (2025). The neuroprotective effects of primary functional components mulberry leaf extract in diabetes-induced oxidative stress and inflammation. J. Agric. Food Chem. 73 (6), 3680-3691. doi: 10.1021/acs.jafc.4c09422, PMID: 39893686 PMC11826978

[B53] Liu X. J. Guo Y. L. (1998). Flora Fungorum Sinicorum, Volume 9: Pseudocercospora (Beijing: Science Press), 1–225.

[B54] Luo L. Zhang X. Wu X. Liu W. Liu J. (2024). Identification of gonatophragmium mori causing mulberry zonate leaf spot disease and characterization of their biological enemies in Guangxi, China. Plant Dis. 108, 162–174. doi: 10.1094/PDIS-04-23-0738-RE, PMID: 37552161

[B55] Maji M. D. Banerjee R. Das N. K. Chakraborty S. Bajpai A. K. (2008). Role of meteorological factors on the incidence of mulberry diseases. Indian J. Sericulture 47, 193–196. doi: 10.5555/20093135008

[B56] Mao Y. Hu Y. Jia Q. Chang M. Zhao M. Sun D. (2025). Protective effects of supercritical CO2-extracted mulberry leaf extract against non-alcoholic fatty liver disease in mice. J. Pharm. Innovation 20, 32. doi: 10.1007/s12247-025-09942-1

[B57] Matić S. Gilardi G. Gullino M. L. Garibaldi A. (2019). Emergence of leaf spot disease on leafy vegetable and ornamental crops caused by *Paramyrothecium* and *Albifimbria* species. Phytopathology 109, 1053–1061. doi: 10.1094/PHYTO-10-18-0396-R, PMID: 30667339

[B58] Mekala V. Muhilan R. Ragul R. K. Rithika C. (2025). “Mulberry leaf disease detection system,” in 2025 3rd International Conference on Intelligent Data Communication Technologies and Internet of Things (IDCIoT) (Bengaluru, India: IEEE), 1886–1892. doi: 10.1109/IDCIOT64235.2025.10915157

[B59] Nguyen H. H. Nguyen T. N. Pham T. P. Le T. T.C. Bui T. K. Jang D. C.. (2024). Leaf position of mulberry (*Morus alba* L.) affects silkworm growth, silk cocoon yield and quality. Vegetos. 38, 1681–1688. doi: 10.1007/s42535-024-00965-6

[B60] Niaz M. Abbas S. Q. Ayesha R. Iftikhar T. (2010). New fungal records on *Morus alba* from Faisalabad Pakistan II. Pakistan J. Bot. 42, 1949–1958.

[B61] Nzioki H. S. Oyoo M. E. Monda E. O. (2020). Diversity and pathogenicity of Pseudocercospora species infecting fruit trees in East Africa. Mycological Prog. 19, 967–975.

[B62] Peris N. W. Lucas N. Miriam K. G. Theophillus M. M. (2012). Field evaluation of mulberry accessions for susceptibility to foliar diseases in Uasin-Gishu district, Kenya. Afr. J. Biotechnol. 11, 3569–3574. doi: 10.5897/AJB11.2495

[B63] Perrotta G. Cacciola S. O. Pane A. Faedda R. (1998). Outbreak of a leaf disease caused by *Pseudocercospora ceratoniae* on carob in Sicily. Plant Dis. 103, 2958–2965. doi: 10.1094/PDIS.1998.82.12.1401C, PMID: 30845479

[B64] Phengsintham P. Braun U. McKenzie E. H. C. Chukeatirote E. Cai L. Hyde K. D. (2013). Monograph of cercosporoid fungi from Thailand. Plant Pathol. Quarantine 3, 67–138. doi: 10.5943/ppq/3/2/2

[B65] Qiu W. F. (1998). Mycology (Beijing: Science Press), 10–11.

[B66] Raja H. A. Miller A. N. Pearce C. J. Oberlies N. H. (2017). Fungal identification using molecular tools: A primer for the natural products research community. J. Natural Products 80, 756–770. doi: 10.1021/acs.jnatprod.6b01085, PMID: 28199101 PMC5368684

[B67] Rohela G. K. Saini P. Syam S. Roy P. Gonal B. Aziz D.. (2025). “Mulberry trees: A sustainable solution for urban forestry and improved air quality,” in Sustainable urban environment and waste management: Theory and practice (Springer Nature Singapore, Singapore), 223–246.

[B68] Romanelli A. M. Fu J. Herrera M. L. (2014). A universal DNA extraction and PCR amplification method for fungal rDNA sequence-based identification. Mycoses 57, 612–634. doi: 10.1111/myc.12208, PMID: 24865530

[B69] Saif Z. Ashraf S. Shah M. D. Mir S. A. Paddar B. A. Nabi A.. (2023). Morpho-cultural, pathological and molecular variability in Phloeospora maculans causing leaf spot of mulberry (Morus species) in India. Mol. Biol. Rep. 50, 8337–8348. doi: 10.1007/s11033-023-08677-x, PMID: 37592179

[B70] Schoch C. L. Seifert K. A. Huhndorf S. Robert V. Spouge J. L. Levesque C. A.. (2012). Nuclear ribosomal internal transcribed spacer (ITS) region as a universal DNA barcode marker for fungi. Proc. Natl. Acad. Sci. United States America 109, 6241–6246. doi: 10.1073/pnas.1117018109, PMID: 22454494 PMC3341068

[B71] Shree M. P. Shivakumar P. Shanthi K. R. Nataraja S. (1997). Influence of powdery mildew and leaf spot disease on the enzyme activity in mulberry varieties. Geobios 24, 98–102.

[B72] Soylu S. Kurt S. Soylu E. M. (2003). First report of phloeospora leaf spot on mulberry caused by Phloeospora maculans (= Cylindrosporium maculans) in the East Mediterranean region of Turkey. Plant Pathol. 52, 415. doi: 10.1046/j.1365-3059.2003.00837.x

[B73] Stielow J. B. Levesque C. A. Seifert K. A. Meyer W. Irinyi L. Smits D.. (2015). One fungus, which genes? Development and assessment of universal primers for potential secondary fungal DNA barcodes. Persoonia 35, 242–263. doi: 10.3767/003158515X689135, PMID: 26823635 PMC4713107

[B74] Teotia R. S. Sengupta T. Das C. (1997). Biochemical changes in the leaves of mulberry (*Morus alba* L.) infected by *Pseudocercospora mori* (Hara) Deighton. Indian J. Sericulture 36, 158–160.

[B75] Walkowiak S. Rowland O. Rodrigue N.. (2016). Whole genome sequencing and comparative genomics of closely related Fusarium Head Blight fungi: *Fusarium graminearum*, *F. meridionale* and *F. asiaticum* . BMC Genomics 17, 1014. doi: 10.1186/s12864-016-3371-1, PMID: 27938326 PMC5148886

[B76] Wang X. D. (2009). Prevention and control of pests and diseases of mulberry (Chengdu: Southwest Jiaotong University Press), 126–129.

[B77] Wang W. F. He N. J. You C. P. Tang X. N. (1994). A preliminary report on the incidence of mulberry leaf blight. J. Jiangxi Agric. Univ. 16, 123–125.

[B78] White T. J. Bruns T. D. Lee S. B. Taylor J. (1990). “Amplification and direct sequencing of fungal ribosomal RNA genes for phylogenetics,” in PCR protocols—A guide to methods and applications. Ed. Innis M. A. (London, UK: Academic Press), 315–322.

[B79] Woudenberg J. H. C. Groenewald J. Z. Binder M. Crous P. W. (2013). Alternaria redefined. Stud. Mycology 75, 171–212. doi: 10.3114/sim0015, PMID: 24014900 PMC3713888

[B80] Xie Y. Li H. Deng Z. Peng H. Yu Y. Zhang B.. (2025). Preparation and characterization of a new food-grade Pickering emulsion stabilized by mulberry-leaf protein nanoparticles. J. Sci. Food Agric. 105, 1080–1090. doi: 10.1002/jsfa.13898, PMID: 39271605

[B81] Zaman W. Ayaz A. Park S. (2025). Integrating morphological and molecular data in plant taxonomy. Pak. J. Bot. 57(4), 1453–1466. doi:10.30848/PJB2025-4(27)

[B82] Zhang Y. J. (1975). A preliminary study on the overwintering mode and pathogenesis of mulberry leaf stain. Sci. Technol. Bull. 7, 16–22.

[B83] Zhang W. Wang D. Hao E. Shi L. Chen H. Zhang W.. (2024). Positive effects and mechanism of mulberry leaf extract on alleviating fatty liver hemorrhagic syndrome in laying hens. Poultry Sci. 103, 103998. doi: 10.1016/j.psj.2024.103998, PMID: 39018653 PMC11305280

[B84] Zhao Q. Shi Y. Wang Y. Xie X. Li L. Fan T.. (2022). Temperature and humidity regulate sporulation of *Corynespora cassiicola* that is associated with pathogenicity in cucumber (*Cucumis sativus* L.). Biology 11, 1675. doi: 10.3390/biology11111675, PMID: 36421389 PMC9687187

