# Supplementary material for: Identification of Pseudocercospora mori as the causal agent of grey leaf spot disease in mulberry (Morus atropurpurea) from various localities in Guangdong Province, China
Source: Front Plant Sci. 2025 Sep 4;16:1648690. doi: 10.3389/fpls.2025.1648690 (PMC12443802; doi:10.3389/fpls.2025.1648690)
Supplement: Supplementary file 1 [file DataSheet1.docx]

**Supplementary material**


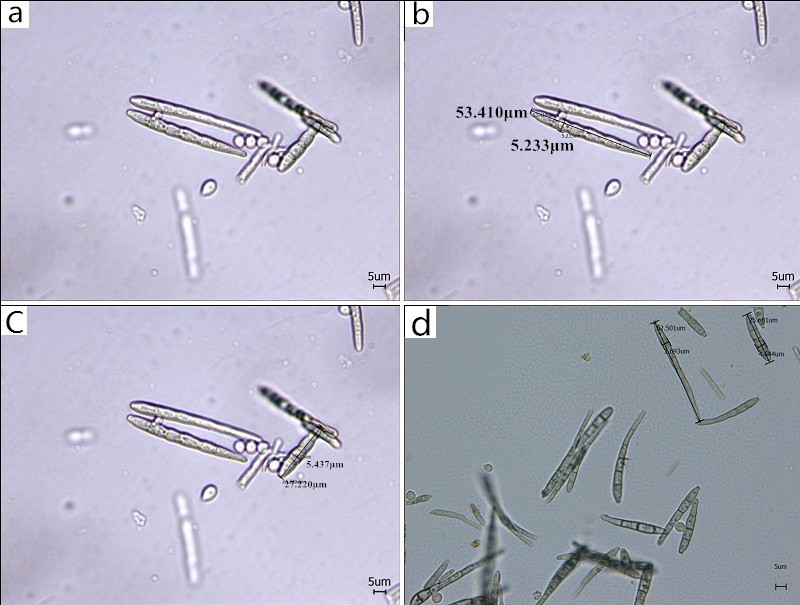


Supplementary figure 1 Measurement of conidia of the pathogen of mulberry leaf spot disease

Note: a is conidia; b is the length and width of the conidia; the scale bar is 5 μm. It can be seen that two different types of conidia exist at the same time, and the length difference between different conidia can reach 1~2 times. In addition, it was observed that the conidia of multiple groups were longer, and the majority of them were found, indicating that most of the conidia on the lesions were produced by hyphae.


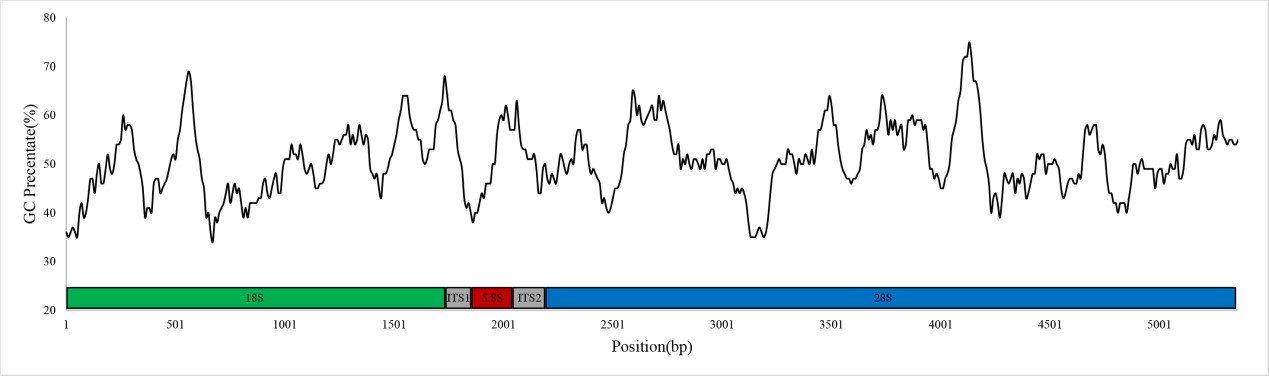


Supplementary Figure 2 The rDNA sequence composition and base GC ratio distribution


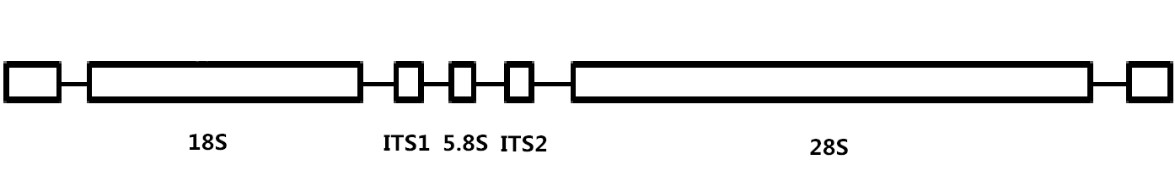


Supplementary Figure 3 Schematic representation of the gene structure of the rRNA of the mulberry leaf spot pathogen
